# Archaea methanogens are associated with cognitive performance through the shaping of gut microbiota, butyrate and histidine metabolism

**DOI:** 10.1080/19490976.2025.2455506

**Published:** 2025-02-05

**Authors:** Andrea Fumagalli, Anna Castells-Nobau, Dakshat Trivedi, Josep Garre-Olmo, Josep Puig, Rafel Ramos, Lluís Ramió-Torrentà, Vicente Pérez-Brocal, Andrés Moya, Jonathan Swann, Elena Martin-Garcia, Rafael Maldonado, José Manuel Fernández-Real, Jordi Mayneris-Perxachs

**Affiliations:** a Department of Diabetes, Endocrinology and Nutrition, Dr. Josep Trueta University Hospital, Girona, Spain; b Nutrition, Eumetabolism and Health Group, Girona Biomedical Research Institute (IDIBGI-CERCA), Girona, Spain; c Integrative Systems Medicine and Biology Group, Girona Biomedical Research Institute (IDIBGI-CERCA), Salt, Spain; d CIBER Fisiopatología de la Obesidad y Nutrición (CIBERobn), Instituto de Salud Carlos III; Madrid, Spain; e School of Human Development and Health, Faculty of Medicine, University of Southampton, Southampton, UK; f serra-hunter program Department of Nursing, University of Girona, Girona, Spain; g Department of Medical Sciences, School of Medicine, University of Girona, Girona, Spain; h Institute of Diagnostic Imaging (IDI)-Research Unit (IDIR), Parc Sanitari Pere Virgili, Barcelona, Spain; i Medical Imaging, Girona Biomedical Research Institute (IdibGi), Girona, Spain; j Department of Radiology (IDI), Dr. Josep Trueta University Hospital, Girona, Spain; k Vascular Health Research Group of Girona (ISV-Girona), Jordi Gol Institute for Primary Care Research (Institut Universitari per a la Recerca en Atenció Primària Jordi Gol I Gorina -IDIAPJGol), Red de Investigación en Cronicidad, Atención Primaria y Promoción de la Salud-RICAPPS- ISCIII Girona Biomedical Research Institute (IDIBGI), Dr. Josep Trueta University Hospital, Girona, Catalonia, Spain; l Research in Vascular Health Group, Girona Biomedical Research Institute (IDIBGI-CERCA), Dr. Josep Trueta University Hospital, Girona, Spain; m Neuroimmunology and Multiple Sclerosis Unit, Department of Neurology, Dr. Josep Trueta University Hospital, Girona, Spain; n Neurodegeneration and Neuroinflammation Research Group, IDIBGI-CERCA, Girona, Spain; o Area of Genomics and Health, Foundation for the Promotion of Sanitary and Biomedical Research of Valencia Region (FISABIO-Public Health), Valencia, Spain; p Biomedical Research Networking Center for Epidemiology and Public Health (CIBERESP), Madrid, Spain; q Institute for Integrative Systems Biology (I2SysBio), University of Valencia and Spanish National Research Council (CSIC), Valencia, Spain; r Laboratory of Neuropharmacology, Department of Experimental and Health Sciences, Universitat Pompeu Fabra, Barcelona, Spain; s Hospital del Mar Medical Research Institute (IMIM), Barcelona, Spain

**Keywords:** Microbiota, cognition, archaea, cognitive flexibility, executive function

## Abstract

The relationship between bacteria, cognitive function and obesity is well established, yet the role of archaeal species remains underexplored. We used shotgun metagenomics and neuropsychological tests to identify microbial species associated with cognition in a discovery cohort (IRONMET, *n* = 125). Interestingly, methanogen archaeas exhibited the strongest positive associations with cognition, particularly *Methanobrevibacter smithii* (*M. smithii*). Stratifying individuals by median-centered log ratios (CLR) of *M. smithii* (low and high *M. smithii* groups: LMs and HMs) revealed that HMs exhibited better cognition and distinct gut bacterial profiles (PERMANOVA *p* = 0.001), characterized by increased levels of Verrucomicrobia, Synergistetes and Lentisphaerae species and reduced levels of Bacteroidetes and Proteobacteria. Several of these species were linked to the cognitive test scores. These findings were replicated in a large-scale validation cohort (Aging Imageomics, *n* = 942). Functional analyses revealed an enrichment of energy, butyrate, and bile acid metabolism in HMs in both cohorts. Global plasma metabolomics by CIL LC-MS in IRONMET identified an enrichment of methylhistidine, phenylacetate, alpha-linolenic and linoleic acid, and secondary bile acid metabolism associated with increased levels of 3-methylhistidine, phenylacetylgluamine, adrenic acid, and isolithocholic acid in the HMs group. Phenylacetate and linoleic acid metabolism also emerged in the Aging Imageomics cohort performing untargeted HPLC-ESI-MS/MS metabolic profiling, while a targeted bile acid profiling identified again isolithocholic acid as one of the most significant bile acid increased in the HMs. 3-Methylhistidine levels were also associated with intense physical activity in a second validation cohort (IRONMET-CGM, *n* = 116). Finally, FMT from HMs donors improved cognitive flexibility, reduced weight, and altered SCFAs, histidine-, linoleic acid- and phenylalanine-related metabolites in the dorsal striatum of recipient mice. *M. smithii* seems to interact with the bacterial ecosystem affecting butyrate, histidine, phenylalanine, and linoleic acid metabolism with a positive impact on cognition, constituting a promising therapeutic target to enhance cognitive performance, especially in subjects with obesity.

## Introduction

The gut microbiota encompasses all the microorganism that reside in our gastrointestinal tract including archaea, bacteria, fungi and virus. In the past decades, its study has gained rising interest due to its involvement in many physiological and pathological processes, in particular in central nervous system disorders.^1^

Targeting the gut microbes offer a new and easier way of facing cognitive impairment, one of the major public threats of our society as the average age is increasing.^2^ A growing body of evidence suggests that the gut microbiota plays a role not only in neurological diseases including dementia or Alzheimer^3,4^ and Parkinson,^5^ but also in psychiatric disorders such as depression,^6,7^ attention deficit hyperactivity disorder,^8^ autism,^9,10^ and cognitive impairment, especially during aging^11^ or metabolic conditions such as obesity.^12,13^ Despite the latter are also risk factors for developing dementia, the target of gut microbiota could help reducing the burden of the whole spectrum of these diseases.

Bacteria are the most abundant and characterized members of the gut microbiota. Some bacterial species have already been associated with neurological and metabolic disorders, while others have been classified as probiotics, mainly Lactobacilli and Bifidobacteria,^14^ given their putative positive effects. However, less information is available about archaea colonizing human gut, primarily due to their physical characteristics and relatively lower abundance when compared with bacteria, which makes their exploration more challenging given the current limitations of available technologies.^15^

*Methanobrevibacter smithii* (*M. smithii*) is a methanogen which constitutes the major species among Archaeas.^16,17^ Although the role of archaea in the development of human diseases is still not clear, there are scarce evidences of their increase abundance in relation with some conditions (see below). Nevertheless, no archaea is currently classified as pathogen, and they are in fact candidate probiotics in some reports.^18^

The levels of methanogens can also be indirectly measured through breath methane emissions; some studies found a correlation between high breath methane emission, constipation and irritable bowel disease related to prevalence of methanogenic gut microbiota.^19,20^ On the contrary, a reduced abundance of methanogens in favour to halophiles archaea have been found in subjects with colorectal cancer.^21^ Other studies reported the absence of *M. smithii* to be associated with malnutrition.^22^

The role of Archaea in weight balance and obesity remains controversial. While higher levels of breath methane have been linked to obesity severity,^23^ the opposite association was found in individuals with functional gastrointestinal disorders.^24^ Similarly, higher levels of *M. smithii* have been associated with increased risk of developing obesity in childhood.^25^ *Zhang et al*. ^26^ also reported higher abundances of *M. smithii* in adult subjects with obesity, although the sample size was limited (n = 9). On the other hand, greater abundance of *M. smithii* was also described among subjects with anorexia.^27^ To the best of our knowledge, limited information is available regarding the involvement of Archaea In neurological disorders. Increased abundance of Methanobrevibacter species were associated with cognitive impairment in women with and without HIV (n = 446).^28^ Another study reported that cognitive impairment in association with higher abundances of Methanobrevibacter was mediated by IL-6 and RANTES in patients with schizophrenia^29^. Increased Methanobrevibacter was also reported in subjects with multiple sclerosis who also display high levels of circulating IL-6.^30^

However, the gut microbiota is composed by communities consisting of species from different domains that interact between each other and with the host. The variation of the abundance of a single species can impact on the others and alter the whole equilibrium both positively or negatively. Due to the complexity of the system composed by different kingdoms and species, and the limitation in isolate and cultivate some of them, little is known about how species from different kingdoms (including archaea) interact and influence each other. Methanogens archaea and bacteria are known to be one of the best examples of syntrophic interaction between species, as the former consume bacterial derived fermentation by-products preventing their accumulation and leading to inhibition of bacterial metabolism.^31^ However, their mutual impact on cognition has been unexplored.

To offer further insights about the possible role of the Archaea, and its interaction with gut bacteria, in relation to cognitive function in middle-aged subjects from general population, we analysed the metagenomic and metabolomic profile of IRONMET cohort (n = 125) and Aging Imageomics cohort (n = 942) by grouping the two cohorts based on high or low levels of *M. smithii* and examining their associations with a panel of neuropsychological tests. Additionally, we performed fecal microbiota transplants (FMT) in mice using donors with either high or low abundance of *M. smithii*. Subsequently, we conducted a behavioral test and metabolomic analyses on the dorsal striatum of the recipient mice.

## Results

### Methanogens archaea are associated with better cognitive performance and specific gut microbial profile.

**Figure 1.**
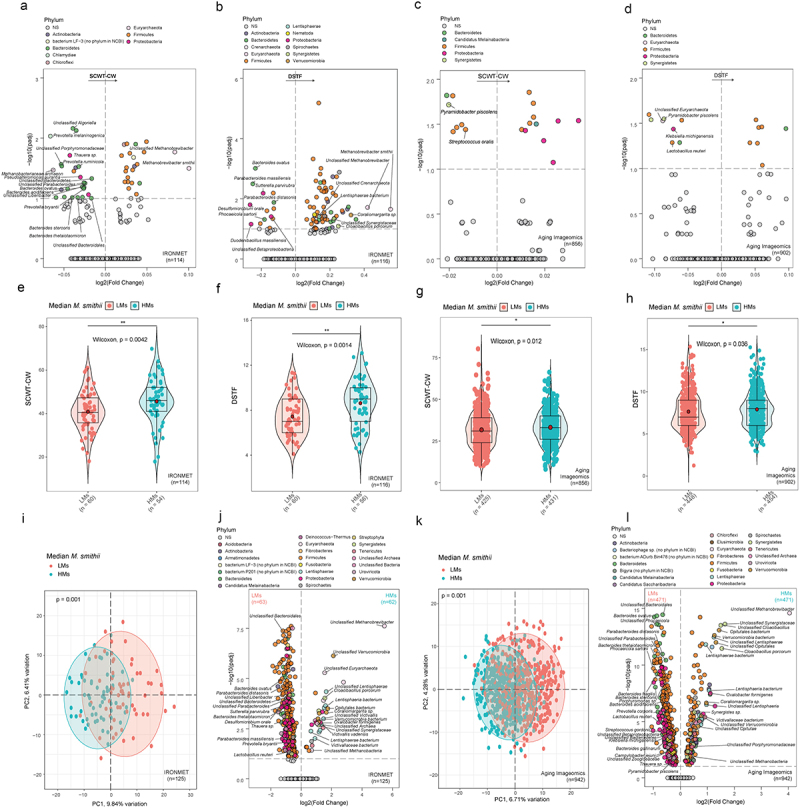
Gut microbial profile and cognitive capabilities associated with *M. smithii groups*. Volcano plot of differential microbial abundance associated with SCWT-CW test (a) and DSTF test (b) in the IRONMET cohort. Volcano plot of differential microbial abundance associated with SCWT-CW test (c) and DSTF test (d) in the Aging Imageomics cohort. Significant species were identified using the ANCOM-BC from shotgun metagenomics data adjusted for age, sex, BMI, and years of education. The log2 fold change of the association with a unit change in the ANCOM-BC-transformed variable values and the log10 p values adjusted for multiple comparisons using a sequential goodness of fit were plotted for each taxon. Significantly different taxa are colored according to phylum. Significance was set at padj<0.1. Violin plots of the SCWT-CW (e) and DSTF (f) tests scores in the IRONMET cohort grouped according to the median of the CLR abundance of *M. smithii* (LMs below median, HMs above median). Significance was assessed using a Wilcoxon test. Red dots represent the mean. #*p*<0.1 *p<0.05, **p<0.01; ***p<0.001. Violin plots of the SCWT-CW (g) and DSTF (h) tests scores in the Aging Imageomics cohort grouped according to the *M. smithii* Median. Significance was assessed using a Wilcoxon test. Red dots represent the mean. #*p*<0.1 *p<0.05, **p<0.01; ***p<0.001. (i)PCA score plot based on clr-transformed shotgun sequencing metagenomic microbial taxonomy data of IRONMET cohort, coloured according to the median of the CLR abundance of *M. smithii* (LMs below median, HMs above median). Differences in the microbiome composition were assessed by PERMANOVA using 999 permutations and Euclidean distances. (j) Volcano plot of differential microbial abundance associated with LMs–HMs groups adjusted for age, sex, BMI and years of education in IRONMET cohort. (k) PCA score plot based on clr-transformed shotgun sequencing metagenomic microbial taxonomy data of IRONMET cohort, coloured according to the LMs–HMs groups. Differences in the microbiome composition were assessed by PERMANOVA using 999 permutations and Euclidean distances. (l) Volcano plot of differential microbial abundance associated with LMs–HMs groups in Aging Imageomics cohort. Significant species were identified using the ANCOM-BC from shotgun metagenomics data adjusted for age, sex, BMI, and years of education. The log2 fold change of the association with a unit change in the ANCOM-BC-transformed variable values and the log10 p values adjusted for multiple comparisons using a sequential goodness of fit were plotted for each taxon. Significantly different taxa are coloured according to phylum. Significance was set at padj<0.1.

To identify the microbial species associated with cognitive function in our discovery cohort (IRONMET, n = 125, Table 1) we used ANCOM-BC methodology adjusting for age, sex, BMI and years of education. We found some archaea species associated with both Stroop color-word test (SCWT-CW)^32^ and digit forward test (DSTF)^33^ (Figure 1a,b; Table S1-S2) the former assessing the executive functions such as inhibitory control and cognitive flexibility and the latter attention and working memory. We identified *Methanobrevibacter smithii* and *Unclassified Methanobrevibacter* as the two species with highest fold change positively associated with both cognitive tests. As expected, we also found a significant association between the clr-transformed *M. smithii* and both cognitive test using Spearman’s correlation (Figure S1A, B). According to these results, we decided to focus on *M. smithii*, being one of the most abundant and described archaea species colonizing human body;^34^ and we segmented our cohort into two groups, this division was calculated using the median of the centred log ratio (CLR) of *M. smithii* obtained from shotgun metagenomic analysis of patients’ stool samples: LMs (below the median, representing lower abundances) and HMs (above the median, representing higher abundances).

**Table 1.** Clinical parameters from the human IRONMET cohort.

	Number (LMs–HMs)	Total Population (n = 125)			
		LMs (Low)	HMs (High)	*P*	
Age	63-62	49.47 ± 10.75	48.10 ± 9.68	0.360	
Sex (females:males)	63-62	48:15	39:23	0.11	
BMI	63-62	36.95 ± 10.04	33.64 ± 10.91	0.07	#
Iron (mcg/dL)	62-62	81.44 ± 26.05	85.82 ± 31.34	0.42	
Glucose (mg/dL)	63-62	97.14 ± 11.52	95.06 ± 12.97	0.5	
Insulin	51-54	20.80 ± 12.75	17.07 13.23	0.075	#
Triglycerides (mg/dL)	63-62	108.75 ± 54.80	103.42 ± 51.06	0.56	
LDL Cholesterol (mg/dL)	63-62	127.78 ± 41.42	117.97 ± 36.19	0.18	
HDL Cholesterol (mg/dL)	63-62	55.92 ± 14.90	58.42 ± 17.48	0.490	
C-reactive protein (mg/dL)	63-59	6.62 ± 8.88	3.58 ± 4.71	0.02	*
Years of education	60-58	12.88 ± 3.32	13.47 ± 3.58	0.3	
Digit Forward	60-56	7.43 ± 1.73	8.62 ± 2.09	0.0014	**
STROOP Color Word	60-54	40.83 9.10	45.63 ± 10.59	0.0042	**

BMI, body mass index. Data are expressed as mean ± standard deviation. We used Wilcoxon test to determine differences between study groups.

Consequently, we evaluated the cognitive capabilities of the two groups and we detected that subjects from HMs displayed higher scores in both SCWT-CW, DSTF tests (Figure 1e,f) even after controlling for age, sex, body mass index (BMI) and years of education (Figure S1C, D).

We then performed a principal component analysis (PCA) to visualize the microbial profile associated with LMs and HMs and we found it to be significantly different (PERMANOVA, *p*=0.001, Figure 1i). To identify the microbial species characterizing these two groups we used ANCOM-BC methodology adjusting for age, sex, BMI and years of education and we found that species belonging to the phyla Verrucomicrobia, Synergistetes and Lentisphaerae were associated with HMs group while species belonging to Bacteroidetes and Proteobacteria phyla and from the order of Lactobacillales were linked to LMs (Figure 1j; Table S3). Interestingly, we found many coincidences between the species positively associated with SCWT-CW and DSTF and HMs group, and the species negatively associated with these cognitive tests and the LMs group (Figure 1a, b, j). In particular, we found a positive association between some species from Verrucomicrobia phylum and the HMs group and also with DSTF score such as *Coraliomargarita sp*.

Likewise, in the Synergistetes phylum the *Cloacibacillus porcorum* and the *Unclassified Synergistacae* positively correlated both with HMs group and DSTF; and in the Lentisphaerae phylum, the *Lentisphaeria bacterium* positively correlated with HMs group and DSTF. Similarly, the species associated with LMs group were also negatively associated with neuropsychological test. Thus, the Proteobacteria *Thaurea sp*. and *Sutterella paraviruba* were negatively associated with SCWT-CW and digit Forward, respectively; *Bacteroides ovatus* was negatively associated with both test and *Bacteroides thetaiotaomicron* was negatively associated with SCWT-CW. These findings suggest that *M. smithii* is associated with a characteristic microbial profile and higher performances in executive functions, inhibitory control, cognitive flexibility, attention and working memory.

To get further insight into the interspecies associations we constructed microbial association networks on the clr-transformed data and we identified *M. smithii* as one of the main hubs both in the whole cohort and in the HMs group (Figure S2). Additionally, we identified a novel cluster of species interacting with *M. smithii*, predominantly from the order Clostridiales, including members of the families *Ruminococcaceae* and *Oscillospiraceae* (Table S4-6). This cluster also included species from the phyla Verrucomicrobia and Lentisphaerae, such as *Lentisphaeria bacterium* and *Opitutales bacterium*, which were also identified using ANCOMBC (Figure S2, Table S4-6).

To validate our results, we performed similar analyses in a second cohort (Aging Imageomics, n=942; Table 2, Figure 1c,d, Table S7,S8). Importantly, after stratifying the participants in the LMs and HMs groups, the results were replicated (Figure 1g–h, k-l; Table S9).

**Table 2.** Clinical parameters from the human Aging Imageomics cohort.

	Number (LMs–HMs)	Total Population (n = 942)			
		LMs (Low)	HMs (High)	*P*	
Age	472-470	67.03 ± 6.87	67.13 ± 7.60	0.760	
Sex (females: males)	472-470	227:245	201:269	0.1	
BMI	472-470	28.20 ± 4.58	27.49 ± 4.09	0.028	*
Glucose (mg/dL)	461-453	112.84 ± 30.80	109.41 ± 24.60	0.24	
Insulin	460-453	11.08 ± 8.43	10.11 ± 6.90	0.015	*
Triglycerides (mg/dL)	461-453	125.97 ± 83.31	117.79 ± 62.71	0.19	
LDL Cholesterol (mg/dL)	456-448	118.53 ± 30.99	118.93 ± 31.47	0.83	
HDL Cholesterol (mg/dL)	461-453	52.99 ± 15.33	52.91 ± 16.01	0.77	
Education LEVEL	467-468	1.46 ± 0.73	1.58 ± 0.75	0.023	*
Digit Forward	453-449	7.64 ± 2.19	7.91 ± 2.09	0.034	*
STROOP Color Word	425-431	31.72 ± 10.77	33.19 ± 9.89	0.012	*

**Note**: BMI, body mass index. Data are expressed as mean ± standard deviation. We used Wilcoxon test to determine differences between study groups.

We found that HMs group were associated with higher cognitive performance (Figure 1g, h). Notably, in both cohorts, we identified a specific gut microbial profile associated with *M. smithii* groups highlighting new possible interaction between methanogens Archaea and species from Verrucomicrobia phylum such as *Opitutales bacterium*, *Coraliomargarita sp*. and *Verrucomicrobia bacterium*, as well as species from the Synergistetes phylum like *Cloacibacillus porcorum* and the Lentisphaerae phylum as *Lentisphaeria bacterium*, *Victivallaceae bacterium*, *Victivallis vadensis* and others (Figure 1j,l; Table S3,S9).

Interestingly, we also found a decrease in BMI (or a trend to decreased BMI) and plasma insulin levels in both cohorts. In addition, in the IRONMET we found significant differences in the levels of the inflammatory marker CRP, with lower inflammation associated with the HMs group (Table 1-2). As we found a significant difference in insulin levels between our two study groups, we repeated the analysis adjusting also for insulin levels, but the results were similar (Figure S3 ; Table S10,S11).

To delve deeper into the differences in BMI between our two study groups (Table 1,2), we performed a Spearman’s correlation between *M. smithii* and BMI. We found that clr-transformed *M. smithii* were negatively correlated with the BMI in IRONMET cohort, while a slightly negative but non-significant correlation was found in Aging Imageomics cohort (Figure S4 A, C). However, in both cohorts, lower CLR are clearly associated with individuals with obesity (Figure S4 B, D).

### HMs group is associated with butyrate, histidine, phenylalanine, linoleic, and bile acid metabolism

**Figure 2.**
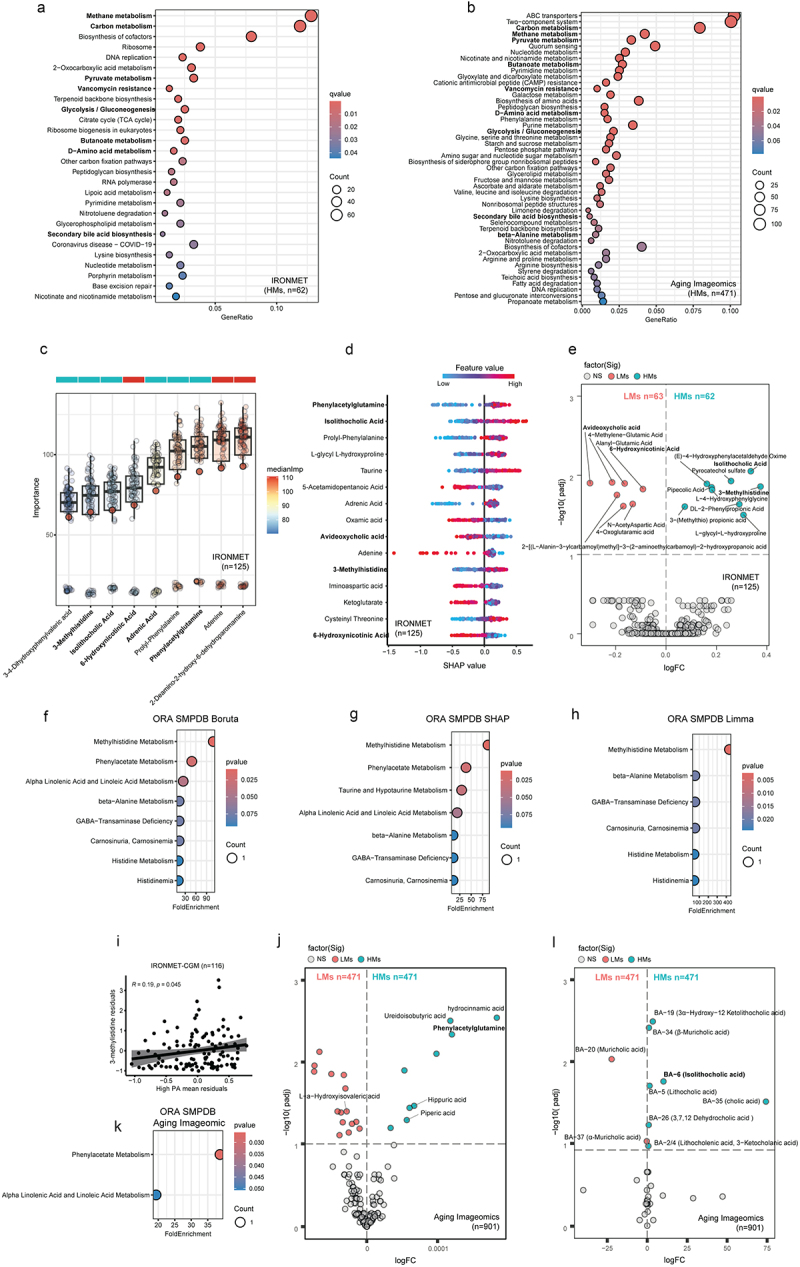
Functional and metabolic profile associated with *M. smithii* groups. Dotplot of KEGG enriched pathways (*q-*value <0.1) from significantly differentially expressed microbial microbial molecular functions associated with the HMs group in IRONMET cohort (a) and in Aging Imageomics cohort (b). Significant KEGG orthologues were identified by ANCOM-BC after adjusting for age, sex, BMI and years of education. Dots are coloured according to the *q*-value. Common pathways are highlighted. (c) Boxplots of the normalized variable importance measure for the metabolites associated with the LMs–HMs groups in IRONMET cohort. Significant metabolites (confirmed) were identified using a machine learning variable selection strategy based on applying multiple random forests as implemented in the Boruta algorithm with 100000 trees, a confidence level cut-off of 0.005 for the Bonferroni adjusted p-values, and a number of features randomly sampled at each split given by the rounded down number of features/3, and controlling for age, sex and BMI. The spot over the boxplot are colored according to the LMs–HMs groups. (d) SHAP summary of the metabolites associated with the LMs–HMs groups in IRONMET cohort. Significant metabolites identified by the machine learning approach are highlighted in bold. Each dot represent an individual sample. The X-axis represents the SHAP value, *i.e*., the impact of a specific metabolite on the prediction of the affinity to LMs or HMs group for a given individual. Colours represent the values of the metabolites levels, ranging from blue (low concentrations) to red (high concentrations). (e) Volcano plot of metabolites associated with LMs–HMs groups in the IRONMET cohort identified with Limma pipeline performing robust linear regression models adjusting for age, sex and BMI. Dotplot of SMPDB-based Over-representation analysis using different set of metabolites associated with the LMs–HMs group identified with Boruta (f), SHAP (g), and Limma (h) in IRONMET cohort. (i) Pearson’s correlation between the 3MH levels and the log transformed mean of intense physical activity residuals adjusted for age, sex, fat free mass and adherence rate in the in IRONMET-CGM cohort. (j) Volcano plot of SMPDB-based Over-representation analysis using the metabolites associated with LMs–HMs groups, identified with Limma pipeline in Aging Imageomics cohort. Dots are coloured according to the p-value. (k) Volcano plot of metabolites associated with LMs–HMs groups in the Aging Imageomics cohort identified with Limma pipeline performing robust linear regression models adjusting for age, sex and BMI. (l) Volcano plot of bile acid metabolites associated with LMs–HMs groups in the Aging Imageomics cohort identified with Limma pipeline performing robust linear regression models adjusting for age, sex and BMI.

To gain further insight in the microbial functionality, we next performed functional analyses by mapping reads to KEGG orthologues and using the ANCOM-BC methodology to identify microbial molecular functions differentially expressed between the two *M. smithii* groups after controlling for age, BMI, sex, and the years of education (Figure S5, Table S12-17). In particular, we observed an enrichment in the microbial pathways associated with energy metabolism, butyrate metabolism, and secondary bile acid biosynthesis in the HMs group in both cohorts (Figure 2a,b, Table S18,S19). We then performed a global plasma metabolic profiling by CIL LC-MS in the IRONMET cohort and used different bioinformatic tools to identify metabolites associated to HMs group after controlling for age, sex, and BMI (Figure 2c-e, Table S20,S21). Using three different methodologies, including the Boruta feature selection machine learning algorithm, SHAP scores on random forest models, and the Limma pipeline with robust regression, we identified that isolithocholic acid, a secondary bile acid derived from the gut microbiota, and 3-methylhistidine were consistently increased in the HMs group compared to the LMs group (Figure 2c, e). Notably, among all metabolites, 3-methylhistidine and isolithocholic acid exhibited the more pronounced increases (strongest fold changes) in the HMs group (Figure 2e). In addition, phenylacetylglutamine, a phenylalanine catabolite formed through the conjugation of glutamine with the gut microbiota-derived phenylacetate, and adrenic acid were two of the metabolites most strongly associated with the HMs group using both Boruta and SHAP analyses. Furthermore, both SHAP and limma methodologies identified avideoxycholic acid, another microbial-derived secondary bile acid, as one of the metabolites most strongly decreased in the LMs group (Figure 2d,e).

We then performed an enrichment analysis using the SMPDB database with metabolites identified through Boruta, SHAP, and limma. Across all methodologies, histidine metabolism, in particular methylhistidine metabolism, emerged as the most enriched pathway, followed by beta-alanine metabolism, which involved 3-methylhistidine (Figure 2f-h, Table S22-24). These findings suggest a potential relationship with muscle turnover, gut microbiota and cognitive functions. To further investigate the link with muscle metabolism and physical activity, we explored this correlation in an independent cohort (IRONMET-CGM, n=116, Table S25). In this cohort, participants wore the Fitbit Charge 3 with an average adherence rate of 71% (min=60%; max=94%). 3-methylhistidine showed a significant positive correlation with the mean duration of the high intensity physical activity, after controlling for age, sex, fat-free mass and adherence rate (Figure 2i).

In addition, the phenylacetate metabolism and the alpha-linolenic and linoleic acid metabolism, involving phenylacetylglutamine and adrenic acid, respectively, were also significantly enriched pathways in the HMs group using the Boruta algorithm and SHAP scores (Figure 2f,g). A KEGG-based enrichment also highlighted an over-representation of the metabolism of histidine and phenylalanine in the HMs group using Boruta and SHAP methodology (Figure S6A-C). However, the most enriched KEGG pathway identified using Boruta, SHAP, and limma analyses was the secondary bile acid metabolism, which involved isolithocholic and avideoxycholic acids (Figure S6A-C). These results align with the metagenomics findings, which also revealed an enrichment of the secondary bile acids biosynthesis and the phenylalanine metabolism in the HMs group (Figure 2a,b).

To further validate these findings, we conducted untargeted plasma metabolomics analysis by HPLC-ESI-MS/MS in negative mode in the Aging Imageomics cohort. Remarkably, robust regression analyses using limma identified once more phenylacetylglutamine as one of most significantly (based on *p*-values) and strongly (based on fold changes) associated metabolites with the HMs group (Figure 2j, Table S26). In addition, consistent with the findings in the IRONMET cohort, the phenylacetate metabolism and the alpha linoleic and linolenic acid metabolism emerged again as the two most significantly enriched pathways identified with SMPDB (Figure 2k, Table S27) while a KEGG-based enrichment highlighted an over-representation of phenylalanine metabolism (Figure S6D, Table S28). Finally, we performed a targeted bile acid metabolomic, and we identified the isolithocholic acid significantly associated with HMs in line with previous findings (Figure 2l, Table S29). It is also worth noting that we could not identify histidine-related metabolites using this untargeted approach, which may account for the lack of consistent findings related to histidine metabolism in this cohort.

### FMT from human donors from HMs group influence cognitive flexibility weight balance and brain metabolomic in recipient mice

**Figure 3.**
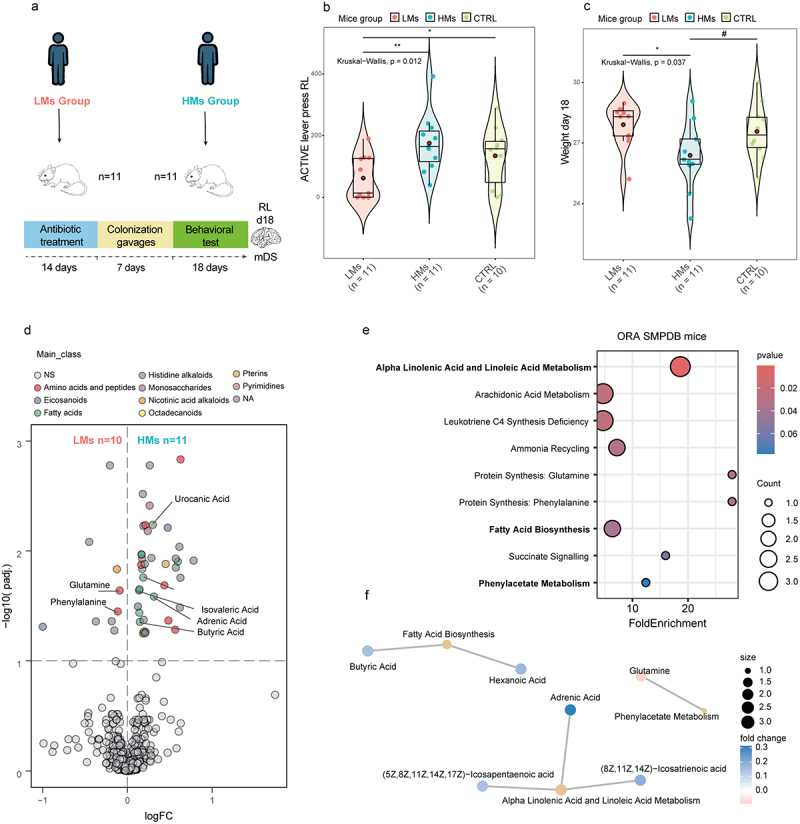
FMT effect on inhibitory control and weight balance in mice. (a) Experimental design for the faecal microbiota transplantation (FMT) study. The microbiota from LMs group, n = 11 and HMs group, n = 11 human donors were delivered to recipient mice pre-treated with antibiotics for 14 d. n = 10 control mice were treated with saline. Violin plots of the reversal learning (RL) tests (b) and weight measurement (c) performed at day 18. Significance was assessed using a Wilcoxon test. Red dots represent the mean. #*p*<0.1 *p<0.05, **p<0.01; ***p<0.001. (d) Volcano plot of metabolites associated with LMs–HMs groups in the dorsal striatum of mice identified with Limma R pipeline performing robust linear regression models adjusting for age, sex, BMI and years of education of donors. (e) Dotplot of SMPDB-based Over-representation analysis from significantly differentially expressed metabolites associated with LMs–HMs groups of mice. Dots are coloured according to the *p*-value. Significant pathways are highlighted. (f) Overenriched pathway network displaying the significant metabolites associated with LMs–HMs groups involved in the fatty acid biosynthesis, phenylacetate metabolism and alpha linoleic and linoleic acid metabolism, colored according to the fold change.

To validate our finding, we performed an FMT in mice from 11 donors belonging to LMs group and 11 donors from HMs (Figure 3a). We found that mice receiving FMT from HMs patients had a better inhibitory control and cognitive flexibility assessed as the number of lever-presses in the inverted active lever in the reversal learning test (RLT) (Figure 3b). Moreover, mice who received FMT from the HMs group exhibit a significant lower weight compared to LMs group, suggesting that the presence of *M. smithii* and its associated microbial profile could also influence weight balance (Figure 3c).

We also performed a metabolomic analysis of the dorsal striatum, controlling for age, sex, BMI and years of education of human donors, to identify metabolites associated with our two study groups. Consistent with the metabolomics findings in human plasma (Figure 2c-e), urocanate, a breakdown metabolite of histidine, emerged as one of the most significant metabolite in the recipient’s mice brain differentiating between the HMs and LMs groups (Figure 3d, Table S30). Also, aligning with the human plasma metabolomic results (Figure 2c,d), adrenic acid was increased in the brains of mice receiving FMT from donors from the HMs group, while phenylalanine and glutamine, the precursors of phenylacetylglutamine, were decreased (Figure 3d, Table S30). Interestingly, we also found an increase in the brain levels of the SCFA butyric acid and isovaleric acid in the HMs group. SMPDB- and KEGG-based enrichment analyses further highlighted the metabolism of alfa linoleic and linolenic acid, involving adrenic acid, and the phenylalanine/phenylacetate metabolism, as two of the most over-represented pathways in the brains of mice from the HMs group (Figure 3e,f, Figure S7, Table S31-32).

## Discussion

Here we report a specific gut microbial profile associated with better cognitive performance in attention, executive function, inhibitory control and cognitive flexibility, identified by grouping our cohorts based on the abundance of *M. smithii*. Surprisingly, most of the species belonging to Bacteroidetes and Proteobacteria phyla previously reported to interact with methanogens archaea^31^ were associated with LMs group. On the other hand, *Oxalobacteraceae* and *Ruminococcacea*e, particularly *Ruminococcus champanellensis*, were associated with HMs. These findings were validated in two independent cohorts comprising participants with distinct features, especially regarding age and obesity status, suggesting the involvement of a mechanism unrelated to these conditions.

The anaerobically break down of macronutrients by bacteria yields products such as SCFA, carbon dioxide and hydrogen; the accumulation of the latter causes the inhibition of this fermentative process with consequences on energy balance. All this can be avoided thanks to methanogens Archaea that consume H_2_ excess and restore fermentation processes and SCFA production.^31,35^ Indeed, we observed that the HMs group was associated with increased expression of microbial genes involved in energy balance and butyrate metabolism.

Butyrate is produced and consumed by gut microbiota, it can be produced by different inter-species interactions with different substrates, as demonstrated by *Bui* et al. ^36^ who proposed an *in vitro* model of co-cultured species including *M. smithii*. Butyrate has been reported to enhance cognitive functions alleviating lead-induced neuroinflammation^37^ and to reduce cognitive impairment caused by increased levels of quinolinic acid in both mice and humans with obesity modulating histone acetylation.^38^

The role of butyrate in energy metabolism, inflammation, weight balance and insulin resistance is well described.^39–41^ This could explain the differences in BMI, insulin and CRP levels we found in our study groups (Table 1-2), with the HMs group having less inflammation and lower plasma insulin levels and BMI. Moreover, butyrate has been found to reduce the incidence of obesity in high-fat diet-fed mice through the stimulation of mitochondrial function in skeletal muscle.^42^ Another evidence of the interplay between gut microbiota, butyrate production and skeletal muscle has been described in another study,^43^ where they found an association between gut microbial synthesis of butyrate and appendicular lean mass in Chinese menopausal woman (n = 482).

In fact, one of the most consistent results from human plasma metabolomics was the association between HMs group and 3MH, which was identified using three different bioinformatics methodologies. This metabolite is derived from histidine in muscle and at minor levels in the small intestine.^44,45^ There is no evidence of a biological effect of this metabolite, once produced it enters the bloodstream and it is eliminated through urine. Nevertheless, higher levels of 3MH have been found correlated with higher executive function scores after 30 min of Physical Activity (Cycling) in a subgroup of participant receiving amino acid (AA) supplementation with essential AA, primarily composed by BCAA.^46^ Another study encountered increased excretion of 3MH after physical activity.^47^ Interestingly, higher abundances and activity of *M. smithii* have been found in professional cyclist compared to amateur.^48^ In line with these evidences, we also observed higher levels of 3-methylhistidine was associated high intensity physical activity in a second validation cohort, supporting a possible role of physical activity together with gut microbiota, and especially *M. smithii*, in enhancing cognitive functions affecting muscle metabolism. The different evidences suggesting the existence of a muscle-gut-brain axis^49,50^ imply a possible role of muscle turnover in the phenotype of our HMs group. Enrichment analyses using differential metabolites between the HMs and LMs groups highlighted the methylhistidine metabolism as the most significant pathways, but also the beta-alanine and histidine metabolism. Beta-alanine is widely used as supplement by athletes as it enhance skeletal muscle performance in anaerobic conditions and could also have effects on cognitive functions.^51^ Similarly, histidine, as a precursor of 3MH, has been linked to cognition.^52–54^ We speculate that higher abundances of *M. smithii* could shape gut microbiota, resulting into an improved energy metabolism increasing butyrate production and enhancing cognitive functions, while 3MH could be a marker of this process, highlighting the role of muscle metabolism, probably linked with physical activity in cognitive functions. Further investigation is needed to determine the cause of *M. smithii* abundances and the role of 3MH in this mechanism.

Our findings were further validated after FMT. Recipient mice receiving microbiota from donors belonging to the HMs group displayed higher cognitive flexibility and lower weight. Moreover, we also performed a metabolomic analysis of the dorsal striatum of these mice, reported to be involved in executive function.^55,56^ Mice receiving the FMT from HMs donors showed increased levels of urocanic acid, a histidine catabolite, in the dorsal striatum. Histidine has been reported to enhance glutamate biosynthesis with consequent effect on cognition^57^. Additionally, urocanate has been described in different biological fluids and has been associated with different bacterial species belonging to Lachnospiraceae and Bacteroides. In particular, lower urocanate to histidine and to glutamate ratios has been encountered in the perirhinal cortex of recipient mice from infant with higher composite cognition.^54^

Other potential mechanisms through which *M. smithii* or associated bacteria could exert effects on cognition could involved the metabolism of bile acids. In fact, we found that the biosynthesis of secondary bile acids was one of the microbial pathways significantly enriched in both the IRONMET and Aging Imageomics cohorts and plasma metabolomics results revealed isolithocholic acid, a microbial-derived secondary bile acid, as one of the most significant metabolites increased in the HMs group using three different bioinformatics approaches. Although secondary bile acids have been associated with cognitive impairment in Alzheimer’s disease^58,59^, little is known about their role in cognitively healthy individuals, and in particular, the impact of isolithocholic acid. However, a recent study found that lower levels of isolithocholic acid were associated with chronic insomnia,^60^ suggesting that its effects on cognition could be mediated through sleep quality. Notably, a co-occurrence network analysis revealed that isolithocholic acid was positively associated with *Ruminococcaceae UCG-002* and *Ruminococcaceae UCG-003*^60^, which is consistent with our network findings identifying a highly connected cluster in the HMs group involving Ruminococcaceae.

Our analyses also revealed a significant role of the phenylalanine metabolism in the HMs group. Specifically, we found that patients with high *M. smithii* levels had higher levels of phenylacetylglutamine in both the IRONMET and Aging Imageomics cohorts. Notably, we also found that FMT from donors with HMs decreased the levels of glutamine and phenylalanine, the precursors of phenylacetylglutamine, in the brains of recipient mice. Higher levels of phenylacetylglutamine have generally been associated with worse cognitive outcomes, particularly executive function and memory, but usually in patients with chronic kidney disease,^61^ receiving dialysis^62^ or Alzheimer’s disease.^63^

We also observed that the HMs group had increased plasma levels of adrenic acid, which is involved in the metabolism of linolenic and linoleic acids, and FMT from HMs donors increased the levels of adrenic acid in the recipient mice brains. In fact, the linoleic acid metabolism was one of the most enriched pathway in the brain of these mice. Adrenic acid is a long-chain n-6 fatty acid (C22:4n-6) synthesized from linoleic acid. Although little is known about this fatty acid and cognition, higher concentrations of adrenic acid in erythrocytes were associated with lower global amyloid-b load in older adults with objective cognitive impairment.^64^ In addition, adrenic acid levels were lower in both phosphatidylethanolamine and phosphatidylserine in the parahippocampal cortex of AD patients compared to age-matched controls,^65^ and was also decreased by a 27% in the plasmalogen phosphatidylethanolamine of frontal cortex from AD patients.^66^

Finally, we also observed increased levels of SCFA in the dorsal striatum of mice receiving microbiota from the HMs group, in particular butyric acid and isovaleric acid. These findings are in agreement with the human metagenomics results, where we found a consistent enrichment of the butyric acid metabolism in the HMs group from two independent cohorts. Animal studies provide strong evidence for the cognitive benefits of SCFA, in particular butyric acid, although studies in humans are still scarce.^67^

### Limitations

Our study has several limitations. First, the human cohort studies included in this work have cross-sectional nature, with no longitudinal data to infer causality, there is no intervention that can confirm the mechanism behind of our findings. Thus, all the results reported in humans are associacions, with a grouping variable based on *M. smithii* CLR abundance, and not direct consequences of its abundance. We recognize also the limitation of fecal microbiota transplantation, as it involves transferring *M. smithii* together with the entire microbial community from human donors.

In summary, we identified a distinct microbial profile characterized by increased abundances of *M. smithii* alongside other bacterial species from the Verrucomicrobia, Synergistetes, and Lentisphaerae phyla, with reduced levels of Bacteroidetes and Proteobacteria. This profile was associated with enhanced cognitive performance, independent of obesity status or age. Additionally, it was linked to an enrichment in butyrate metabolism and elevated plasma 3-methylhistidine levels. These findings suggest that archaeal species, particularly *M. smithii*, play a critical role in modulating gut microbiota, changing bacterial functionality and potentially influencing the muscle-gut-brain axis. This presents a novel approach to combat cognitive impairment, particularly in the elderly and individuals with obesity.

Further research is necessary to clarify the causal relationship between Archaea and other gut commensals, their connection to muscle metabolism, and whether cognitive benefits arise from the presence of specific species or the absence of others. Nonetheless, the manipulation of *M. smithii* could lead to a reorganization of gut microbial communities that directly or indirectly enhance cognitive function. This presents a promising strategy for addressing both gut dysbiosis and cognitive decline. Overall, this study highlights the potential of archaeal species to beneficially influence gut microbiota, opening new avenues for research into their role in mental and overall human health.

## Materials and methods

### Human cohorts

#### IRONMET cohort (n=125)

Human subjects were recruited from the IRONMET cohort, a case–control study aimed at exploring the relationships between glucose metabolism, brain iron levels, cognitive performance, and gut microbiota composition. The cohort included n=125 subjects with (n=71) and without obesity (n=54) aged 27–66 y (38 males and 87 females) (Table 1). Exclusion criteria included severe systemic diseases unrelated to obesity, conditions with inherent inflammatory activity, clinical signs or symptoms of infection within the past month, use of antibiotics, antifungals, or antivirals in the previous 3 months, pregnancy or breastfeeding, significant eating disorders, major psychiatric history, excessive alcohol consumption (≥40 g/d for women or 80 g/d for men), and a history of brain injury or trauma. All subjects gave written informed consent, validated and approved by the Ethics committee of the Hospital Dr Josep Trueta (CEIm Code 2015.111).

#### The Aging Imageomics (n=942)

The Aging Imageomics Study (Table 2) is an observational study that erolled participants from the province of Girona (Spain). The subjects sourced from two independent cohorts: Maturity and Satisfactory Ageing in Girona study (MESGI50 study) and the Improving interMediAte RisK management study (MARK study). Samples and data from participants included in this study were provided by the IDIBGI Horizontal Aging Program and the IDIBGI Biobank from the Aging Imageomics Study. They were processed following standard operating procedures with the appropriate approval of the Ethics and Scientific Committees. The Aging Imageomics Study is an observational study including participants of the province of Girona (Northeast Catalonia, Spain). Detailed description of the cohort can be found here.^68^ Briefly, the Aging Imageomics Study aimed to identify biomarkers of human aging by examining imaging, biopsychosocial, metabolomic, lipidomics, and microbiome variables. The participants were selected based on the following criteria: age 50 y or older, residing in the community, no infection in the past 15 d, and absence of contraindications for undergoing the magnetic resonance imaging (MRI). The ethics committee of the Dr. Josep Trueta University Hospital approved the protocol of the study and all participants gave informed consent (CEIm Code 2017.146).

#### IRONMET-CGM (n=116)

Patients were recruited as part of the IRONMET CGM, a case–control study design to investigate the associations between glucose metabolism, cognitive function, brain iron content and gut microbiota composition. The cohort included 116 subjects (24–67 y, 34 males and 82 females, BMI 32.76 ± 10.04 kg/m^2^, Table S25) with obesity (n=57, BMI>30 kg/m^2^) and without obesity (n=59, BMI<30 kg/m^2^). Exclusion criteria included severe systemic diseases unrelated to obesity, conditions with intrinsic inflammatory activity, clinical signs or symptoms of infection within the past month, chronic use of anti-inflammatory treatments (steroidal and/or non-steroidal), use of antibiotics, antifungals, or antivirals in the previous 3 months, pregnancy or breastfeeding, significant eating disorders, major psychiatric history, excessive alcohol consumption (≥40 g/d for women or 80 g/d for men), disturbances in iron balance, and a history of major psychiatric conditions. Written informed consent was obtained from all subjects, validated and approved by the Ethics committee of the Hospital Dr Josep Trueta (CEIm code 2017.139, https://clinicaltrials.gov/ct2/show/NCT03889132).

### Neurophysiological assessment

*Digit Forward test (DSTF)*, a subtest of the Digit Span tests and part of the Wechsler Adult Intelligence Scale-III (WAIS-III)^33^ measures general intellectual function. In the DSTF test, the examinee repeats a sequence of numbers in the same order they were presented. A higher score reflects a better attention and working memory function.

*The Stroop Color-Word Test (SCWT-CW)* (Golden version) was used to assess attentional flexibility, selective attention, inhibition, and processing speed. This version consists of three tasks: 1) read letter names 100 printed in black ink so fast as possible, 2) read green, blue, and as soon as you can. Name the ink color of the “XXX” symbol 100 printed in red, or 3) Name the ink color of the letter name 100 where the ink color does not match the word (e.g. “Red” printed in blue ink), than to read the title word Test time is 45 s, last completed items are also recorded, resulting in three points: one for each task (“W”, “C”, and “WC”). Scores from the third task (Word Color) were used in this analysis. The experiment was conducted according to the standardized protocol outlined in the manual.^32^

All tests were displayed as raw scores.

### Sample collection and processing

Faecal samples were collected either at home or in the hospital using a sterile container. If collected at home, the samples were kept at ± 4°C and delivered to the hospital within 4 hours. Upon arrival, samples were processed immediately. The QIAamp DNA mini stool kit (Quiagen, Courtaboeuf, France) was used to extract total DNA from frozen human feces, with DNA concentration measured fluorometrically using the Qubit® Fluorometer 3.0 (Thermo Fisher Scientific, Carlsbad, CA, USA), following the manufacturer’s instructions. For blood samples, blood was collected in EDTA tubes, and plasma was separated by centrifuging at 1600 g for 15 min at 4°C. The supernatant was then collected in a sterile tube and stored at −80°C.

### Clinical diagnositcs

The analyser Cobas® 8000c702 (Roche Diagnostics, Basel, Switzerland) was used to assess lipids profile, high-sensitivity C-reactive protein (hsCRP) and fasting plasma glucose (FPG). All assays were performed following the manufacturer’s protocol on plasma samples.

### Genomic DNA isolation from fecal samples and whole-genome sequencing

#### IRONMET cohort

The QIAamp DNA mini stool kit (QIAGEN, Courtaboeuf, France) was used to isolate total DNA from frozen human stool samples. Qubit 3.0 Fluorometer (Thermo Fisher Scientific, Carlsbad, CA, USA) was employed for quantification of DNA using 0.2 ng/μl (1 ng for each sample) to generate a library for high-throughput sequencing adopting the Nextera DNA Flex Library Prep kit (Illumina, Inc., San Diego, CA, USA) following the manufacturers`instruction.

The NextSeq 500 sequencing system (Illumina) at the facilities of the Sequencing and Bioinformatic Service of the FISABIO (Valencia, Spain) was used to perform sequencing using 2 × 150-bp paired-end chemistry.

#### The Aging Imageomics (n=942)

Total DNA was obtained from frozen human stool using PowerSoil DNA extraction kit (MO BIO Laboratories). A 400–500 ng of total DNA was employed to prepare Illumina sequencing library using the Illumina DNA Prep kit (Illumina). The library was evaluated with a TapeStation Highly Sensitive DNA kit (Agilent Technologies). Qubit (Invitrogen) was used in the quantification of the library. Equimolar amounts of validated libraries were pooled, then sequenced as a paired-end 150-cycle run on an Illumina NextSeq2000. Raw readings were filtered for QV > 30 using an in- house python script.

Before analyzing microbial taxonomic and functional diversity, the FASTQ output files of the samples from all cohorts were pre-processed using fastp^69^, a FASTQ data pre-processing tool for quality control, trimming of adapters, and quality filtering. Clean reads were mapped against the Homo sapiens genome database (GRCh38.p13) using Bowtie2^70^ to remove reads from human origin. Unmapped reads were run using the SqueezeMeta v1.3.1^71^ using the co-assembly mode to pool all samples in a single assembly. Contigs assembly was carried out with megahit^72^ the mapping of reads in contigs was performed with Bowtie2. Prodigal^73^ was employed for ORFs prediction, Diamon^74^ for ORF search and alignment against the GenBank nr database for taxonomic assignment, and the KEGG database for functional annotation.

### Untargeted quantification of plasma metabolites in the IRONMET cohort and dorsal striatum of recipient mice

Prior to each procedure, samples were randomized to minimize potential technical variability from sample preparation and instrument rotation. The randomized samples were then used for subsequent preparation and analysis.

For mice brain tissue samples, metabolite extraction was used and six ceramic beads were added to the sample vials together with 500 μL of a solution (4:1 v/v) of LC-MS grade MeOH/water and then homogenized at 4.5 m/s for 15 s. Subsequently, the homogenized were kept at −20°C for 10 min and then spinned at 15,000 g for 10 min. Finally, all the solvent was moved to a new vial and dried while the sample extract were re-homogenized in 30 μL of LC-MS grade water. NovaMT Sample Normalization kit was used to evaluate the total concentration of the samples and according to that the sample concentration was adjusted with water in order to reach 8mM.

To prepare and aliquot the combined samples, each individual sample was vortexed and spinned at 15,000 g for 1 min. The supernatant was divided into four aliquots for labeling procedures, backup and pooled sample preparation. For the combined samples, 30 μL of samples were pooled and mixed thoroughly from each individual sample and mixed thoroughly to create a reference pool.

Each human sample was centrifuged, subsequently 90 μL of LC-MS grade methanol was incorporated to precipitate the proteins. The methanol extract was incubated at 20°C for 30 min to dry completely, and then it was stored temporarily in at -80°C until labeling.

#### Chemical isotope labeling

Before the labeling, 25 μL and 9.5 μL (for human and mice samples respectively) of LC-MS grade water was added to the aliquot for amine-/phenol-labeling. The SOP present in the kit were rigorously adhered to in the labeling protocol. Shortly, the sample was mixed with 12.5 μL and 6.25 μL (for human and mice samples respectively) of the buffer Reagent A and 37.5 μL and 18.75 μL (for human and mice samples respectively) of Reagent B, consisting in ^12^C2-labeling (for the individual and combined sample) or ^13^C2-labeling (for the combined sample) and mantained at 40°C for 45 min. Subsequently, 7.5 μL and 3.75 μL (for human and mice samples respectively) of quenching Reagent C were included to neutralize the excess of labeling reagent and kept at 40°C for 10 min more. Lastly, 30 μL and 15 μL (for human and mice samples respectively) of the pH regulating Reagent D was incorporated.

Before the labeling, 25 μL and 9.5 μL (for human and mice samples respectively) of LC-MS grade ACN/water (3:1 v/v) was incorporated to the aliquot of sample for carboxyl-labeling. The SOP present in the kit were rigorously adhered to in the labeling protocol. In short, the sample was mixed with 10 μL and 5 μL (for human and mice samples respectively) of the catalyzing Reagent A and 25 μL and 12.5 μL (for human and mice samples respectively) of Reagent B consisting in ^12^C2-labeling (for the individual and combined sample) or ^13^C2-labeling (for the combined sample). After that the samples were vortexed and spinned down and then the mixture was kept at 80°C for 60 min. Finally, 7.5 μL and 20 μL (for human and mice samples respectively) of quenching Reagent C were included to neutralixe the excess of labeling reagent and kept at 80 °C for 30 minutes more.

Equal volume of the ^12^C2-labeled individual sample and ^13^C2-labeled reference sample was then combined. The mixture was ready to be analyzed by LC-MS. Before to anlyse the entire sample set with LC-MS, the quality control (QC) sample was prepared mixing equal volumes of a ^12^C-labeled and a ^13^C-labeled combined sample.

#### LC-MS analysis condition

The LC-MS analysis adhered strictly the SOP (i.e., Rapid LC-MS Analysis for HP-CIL Metabolomics Platform). Every 20 sample runs QC samples were injected to monitor instrument performance.

The LC analysis was performed with The Thermo Scientific Vanquish LC linked to Bruker Impact II QTOF Mass Spectrometer while chromatographic separation with a Agilent eclipse plus reversed-phase C18 column (150 × 2.1 mm, 1.8 μm particle size). The mobile phase was composed of 0.1% formic acid in water (v/v) (A) and 0.1% formic acid in acetonitrile (v/v) (B). The column temperature was maintained at 40°C, and gradient elution was performed with a flow rate of 400 μL/min starting at 25% B increasing to 99% B over 10 min, maintained until 15 min, and finally returning to 25% B at 15.1 min until 18 min. Mass range = m/z 220–1000 and acquisition rate = 1 hz.

#### Data processing and cleansing

LC-MS data from 2-channel analysis (LC-MS data, including QC, in each channel) were first exported to.csv file with Bruker DataAnalysis 4.4. The exported data were uploaded to IsoMS Pro 1.2.20. After Data Quality Check, Data Processing was performed. Parameters used for data processing are:

- Minimum m/z=220
- Maximum m/z=1000
- saturation intensity=20000000
- retention time tolerance=9 seconds
- mass tolerance=10ppm

Peak pairs without data present in at least 80.0% of samples in any group were filtered out. Data were normalized by Ratio of Total Useful Signal.

#### Metabolite identification

The metabolites were identified with high-confidence searched against a labelled metabolite library (CIL Library) based on accurate mass and retention time. The CIL Library contains more than 1,500 experimental entries. Or linked identity library (LI Library) includes over 9,000 pathway-related metabolites, providing high-confidence putative identification results based on accurate mass and predicted retention time matches. The parameters used for metabolite identification are below.

- Retention Time Tolerance for CIL Library ID = 10 seconds
- Retention Time Tolerance for LI Library ID = 75 sexonds
- Mass Tolerance for CIL Library ID = 10 ppm
- Mass Tolerance for LI Library ID = 10 ppm
- Mass Tolerance for Mass-Based Database ID = 10 ppm

### Untargeted quantification of plasma metabolites in the Aging Imageomics cohort

#### HPCL-ESI-MS/MS metabolomics analyses

For non-targeted metabolomics analysis was used an established protocol^75^ Shortly, 30μl of cold methanol (containing phenylalanine-C13 as an internal standard) were added to 10 μl of each plasma sample then mixed by vortexing for 1 minute, and incubated at −20°C for 1 h. After homogenization withFastPrep-24™ (MP biomedicals) the samples were incubated in a roker at 4°C overnight. Following incubation, the samples were centrifuged at 12,000g for 3 min, after which the supernatant was collected and filtered through a 0.2 μm Eppendorf filter. Two microliters of the extracted sample were injected onto a reversed-phase column (Zorbax SB-Aq 1.8 μm, 2.1 × 50 mm; Agilent Technologies) paired with a pre-column (Zorbax-SB-C8 Rapid Resolution Cartridge, 2.1 × 30 mm, 3.5 μm; Agilent Technologies) maintained at 60°C. The flow rate was set at 0.6 mL/min. Solvent A consisted of water with 0.2% acetic acid, and solvent B was methanol with 0.2% acetic acid. The gradient started at 2% solvent B, ramping up to 98% over 13 min, held at 98% for 6 min, and concluded with a 5-min post-run equilibration.

Data were acquired in negative electrospray ionization (ESI) mode, using time-of-flight (TOF) in full-scan mode across a mass range of 50–3000 m/z, with an extended dynamic range of 2 GHz. Nitrogen was employed as the nebulizer gas at a flow rate of 5 L/min and temperature of 350°C. The capillary voltage was set at 3500 V, and the scan rate was 1 scan/s. For continuous mass calibration, a separate nebulizer introduced reference mass compounds (121.050873 and 922.009798) at a low level (10 L/min). Data collection and analysis were performed using MassHunter Data Analysis Software and MassHunter Qualitative Analysis Software (both from Agilent Technologies, Barcelona, Spain). The molecular features representing different co-migrating ionic species for each molecular entity were extracted using the Molecular Feature Extractor algorithm (Agilent Technologies, Barcelona, Spain).

We included samples with a minimum of two ions, excluding those with multiple charge states. Compounds from different samples were aligned using a retention time window of 0.1% ± 0.25 min and a mass accuracy window of 20.0 ppm ± 2.0 mDa. Only compounds detected in at least 50% of the samples within a group were selected, with adjustments made to minimize individual bias.

### Targeted quantification of plasma bile acid in the Aging Imageomics cohort

A targeted LC-MS/MS technique employing multiple reaction monitoring (MRM) transitions tailored for specific bile acids was modified from an earlier validated protocol for measuring bile acids in human plasma.^76^ The approach utilized 45 bile acid reference compounds and 17 deuterated bile acids as internal references (IS). Calibration curves were constructed over a concentration range of 5 nM to 8000 nM.

#### Sample preparation

Samples were processed and analyzed across multiple analytical batches. For each batch, plasma samples, calibrants, quality control (QC) samples, and blank samples were prepared. Plasma samples were processed by mixing a 50 µL aliquot with 10 µL of internal standard (IS) mixture and 150 µL of ice-cold methanol. The mixture was vortexed for 15 min, incubated at -20 °C for 20 min, and centrifuged at 13,000 g and 4°C for 15 min. Solvent blanks were created by substituting an equivalent volume of plasma with a 2:2:1 (v/v) mixture of water, isopropyl alcohol (IPA), and acetonitrile (ACN). QC samples were prepared using low, medium, and high concentration standards. The supernatants from centrifuged plasma samples, blanks, calibrants, and QCs were transferred to a 96-well plate and agitated for 15 min. A 5 µL aliquot of each prepared sample was then injected into the LC-MS/MS system for analysis.

#### LC-MS/MS analysis

UPLC setup: Waters Xevo TQ-XS triple quadrupole mass spectrometer (MS) coupled to Acquity UPLC with BEH C8 column (100 mm × 2.1mm, 1.7 µm) was used to execute bile acid quantification. The mobile phase consisted of Phase A – water:acetonitrile (10:1, v/v) containing 0.007% ammonium acetate and 0.0318% acetic acid (pH ~ 4.16); Phase B - acetonitrile:isopropyl alcohol (1:1, v/v). The gradient elution started at 10% B with a flow rate of 0.6 mL/min, held for 0.1 min, and then linearly increased to 35% B over 9.25 min. It further ramped up to 85% B at 11.25 min (0.65 mL/min) and reached 100% B by 11.8 min, with the flow rate increasing from 0.8 mL/min to 1 mL/min between 11.8 and 13.05 min. This condition was maintained until 13.1 min, followed by re-equilibration at 10% B from 13.2 to 18 min as the flow rate decreased to 0.6 mL/min. The column temperature was maintained at 60°C.

MS Conditions: The mass spectrometer operated in negative electrospray ionization mode (-ESI), using nitrogen as the desolvation gas and argon as the collision gas. Instrument settings included a capillary voltage of 1.57 kV, cone voltage of 60 V, source offset of 30 V, desolvation temperature of 600°C, source temperature of 150°C, desolvation gas flow at 1000 L/hr, cone gas flow at 150 L/hr, and nebulizer gas pressure of 7.0 bar. Details on compound-specific MRM transitions and associated collision energy (CE) values are available in the original method.^76^

#### Calibration and quantification

Waters MassLynx v4.2 software with the TargetLynx package was used to process the data. Bile acid concentrations were determined by calculating the integrated peak area ratios of bile acids to their corresponding internal standards, applying a linear regression model with a 1/x weighting factor. Calibration curves were validated for linearity (R^2^ ≥ 0.99) and accuracy (within ±20% of the QC sample values). Concentrations of 45 bile acids and all internal standards for all subject samples were consolidated into a single matrix for downstream analysis. Pre-processing steps, such as missing value imputation, normalization, scaling, and log-transformation, were performed as needed. The processed dataset was subsequently used for univariate and multivariate analyses to identify significant patterns and correlations.

### Physical activity monitoring in IRONMET-CGM cohort

The Fitbit Charge 3 (FC3) (Fitbit, Inc. https://help.fitbit.com) was used to monitor physical activity. The FC3 is a commercial multi-sensor device designed for activity and sleep tracking. It employs a three-axis accelerometer to capture movement data, including step counts, and provides information on various activity metrics such as intensity, duration, and frequency. Data from the FC3 was synchronized with the Fitbit Mobile App on an LG G6 (LGM-G600L) via Bluetooth, allowing real-time monitoring of physical activity and sleep patterns. Participants wore the FC3 on their wrists, and it measured multiple physical activity parameters, including minutes spent in high-intensity activity, total steps, and energy expenditure. Additionally, the device uses optical heart rate sensors and altimeters to track heart rate and elevation changes, which provide further insight into the intensity and type of physical activity.

### Statistical analysis

Statistical analyses were performed using R (v 4.1.1). First, normality and homogeneity of variances were tested visually and using Shapiro–Wilk test of normality. The Wilcoxon test was used to compare differences between groups. Spearman’s analysis was used to determine the correlation between quantitative variables.

#### Metagenomics analysis

Microbial taxa and molecular functions were filtered so that only those with >10 reads in at least 10% samples were selected. Differential abundance analyses for taxa and KEGG orthologues associated with LMs–HMs group or cognitive test scores were calculated using the ANCOM-BC methodology.^77^ We adjusted the models for age, sex, BMI, and years of education.

The Sequential Goodness-of-Fit,^78^ method executed with R package “SgoF”, was used to adjust the p-values for multiple comparisons; this methods boost their power with augmented number of tests contrarly to FDR methods which reduce their statistical power as the number of tests grow.

When managing a high volume of tests and a small sample size, a common scenario in large omics datasets, SGoF demonstrated to work particularly better than FDR methods. KEGG functional enrichment or over-representation analysis of differentially expressed genes was performed using the clusterProfiler R package,^79^ based on hypergeometric distribution. For multiple testing correction control, clusterProfiler q-values were estimated (Storey correction) and significance was set at q-value <0.1.^80^

We performed a network analysis using NetCoMi R package^81^ using Spearman’s correlation to compute the associations between taxa and setting a threshold of 0.5 for significant interaction.

#### Metabolomics analysis

To identify metabolites associated with LMs–HMs group, we employed a machine learning variable selection approach on the ranked residuals of the metabolites after controlling for age, sex, and BMI. Metabolomics data was normalized by the ratio of total useful signal. Then, we used a multiple random forest-based algorithm as implemented in the Boruta R package.^82^ This method is composed by four steps: randomization, model building, statistical testing, and iteration until the status of all features is decided.

A two-tailed binomial test with Bonferroni correction was adopted to identify important features and evaluate whether they are relevant (significantly higher, confirmed) or not (significantly lower, rejected). The algorithm was run with a confidence level cut-off of 0.005 for the Bonferroni adjusted P-values and 100000 trees.

Due to their nature, ML models are complex and difficult to interpred and often defined as “black-boxes”. To evaluate the influence of every selected metabolite for every subject in the model, we applied complex tools founded on game-theory to calculate the precise Shapley Additive exPlanations (SHAP) values.^83^ The precise estimation of SHAP scores ensures that explanations remain consistently accurate and locally relevant. The importance of a particular score in a particular metabolite is implemented by SHAP values through the comparison between the presence and absence of the metabolite in the model for every subject. A specific characteristic can have distinct SHAP scores for diverse subjects based on the interplay with other traits for every single individual. To estimate and plot the SHAP scores we used R packages “treeshap” and “SHAPforXGBoost” were used.

We also conducted a parallel analysis using the “limma” R package,^84^ performing robust linear regression models controlling for age, sex and BMI performing complete case analyses applying the “lmFit” with the selection of “robust” method to reduce the effect of outliers. The Sequential Goodness of Fit,^78^ method executed with R package “SgoF”, was used to adjust the p-values for multiple comparisons, establishing an adjusted p-value (padj.)<0.1 as a threshold for significance.

### Mice faecal microbiome transplantation (FMT) experiments

All animal procedures were executed following the guidelines of the European Communities Council Directive 2010/63/EU ruling research with animals and were accepted by the local ethical committee (Comitè Ètic d´Experimentació Animal – Parc de Recerca Biomèdica de Barcelona, CEEA-PRBB). For this FMT experiment, we used thirty-two wild-type C57BL/6J male mice. After the animals arrived at the animal facilities, they were let to adapt to housing conditions (12-h cycle of reversed light/dark, 08:00 AM lights off) in the course of 5 d. Mice were kept in single housing with regulated conditions of temperature (21±1 °C) and humidity (55 ± 10%). During the whole study, animals had access to food and water ad libitum. The Operant behavior testing has always been conducted throughout the first hours of the dark phase of the reversed light/dark cycle. The body weight of mice was monitored for the duration of all the experiments.

#### Preparation of fecal suspensions

Feces collected from human donors were suspended in a autoclaved PBS solution containing cysteine (1g/L), used as an antioxidant to improve the viability of anaerobes, and sterile glycerol (10% v/v) to protect microbial cells from damage induced by freezing; homogenized manually and vortexed. Then, they were filtered through a 70 µm filter, aliquoted, and stored at −80ºC. Fecal suspensions were administered to recipient mice by oral gavage at a final volume of 200 µL per mouse.

#### Experimental design FMT Study

After the adaptation period to the habitat environment, animals were joined for mean weight and split into Control (n=10) and Transplant (n=22) groups.

The depletion of gut microbiota in the transplant group mice was achieved adding a cocktail of antibiotic in drinking water served ad libitum during 14 d. The cocktail of antibiotics included imipenem (250 mg/L), ampicillin (1 g/L), vancomycin (400 mg/L), metronidazole (1 g/L) and ciprofloxacin HCl (250 mg/L). After this period of antibiotic intake, animals underwent a 72h wash out period followed by a colonization consisting in daily oral gavage of donor microbiota (200 μL) for the following 3 d. The faecal samples of subjects belonging to LMs group (n=10) and HMs group (n=11) were used to obtain the donor microbiota. To reinforce the effect of donor colonization, two booster inoculations per week were given throughout the whole duration of the experiment. Identical procedure was carried out with control animals with the difference of getting oral gavage of 200 μL of saline solution (0.9% NaCl) rather than donor microbiota.

The food self-administration procedure (see below) began 10 d post the first oral gavage and continued for the following 18 d.

Mice were sacrificed 2 d post the self-administration procedure, the brains were quickly removed, and the brain tissues were then frozen by immersion in 2-methylbutane surrounded by dry ice, and stored at -80°C for further metabolomic analysis.

#### Food self-administration procedure

Operant responding was conducted using mouse operant chambers (Model ENV-307A-CT, Med Associates, Georgia, VT, USA). The start of every food self-administration period was marked by a house ceiling light activated throughout the first 3 seconds of the session. Every period lasted 60 min (25/10/25) during wich regular-flavoured pellets were supplied. The food self-administration session comprised two pellet periods of 25 min each altered by 10 min of pellet-free period in which no pellet was given. The beginning of this 10 min period was marked by the illumination of the entire operant chamber. During the pellet period, the pellet was delivered after an active response paired with a light stimulus (cue light) followed by 10 s of time-out period during which no pellet was dispensed and the cue light was off. The press of the inactive lever provided no pellet reward. All responses on both active and inactive lever were recorded included during the time-out period. For the first 7 d of the operant conditioning session, the reward was set to fixed ratio 1 (FR1) of reinforcement (one active lever-press resulted in a delivery of one pellet). After the FR1 phase, the FR was increased up to 5 lever-presses in order to obtain one reward during the following 8 d. To achieve the operant responding, the following criteria were set: (1) minimum of 75% response on the active lever; (2) obtain at least 5 pellet each session (5 and 25 active lever presses in FR1 and FR5, respectively).

At the end of every period mice were sent back to their cells.

#### Cognitive flexibility

8 d post the FR5, mice underwent to 2 periods of reversal learning (RL) during which active and inactive levers were switched. Thus, the inactive lever during FR1 and FR5 phases turned into the active and the other way around. Higher scores of cognitive flexibility was determined by the number of lever press inverted active lever (inactive during FR phases).

#### Statistical analysis

All statistical analysis was conducted with R (v 4.1.1). Overall differences between study groups were assessed using the Kruskal–Wallis test. The Wilcoxon test was used to compared differences between groups. #*p*<0.1 *p<0.05, **p<0.01; ***p<0.001.

## Supplementary Material

Supplemental Material

## Data Availability

The datasets that support the findings of the study are available from the corresponding authors upon reasonable request. The raw metagenomics sequence data derived from human samples in the IRONMET and Aging Imageomics cohorts have been deposited in the European Nucleotide Archive (ENA) under the project numbers PRJEB39631 and PRJEB52682, respectively.

## References

[1] Cryan JF, O’Riordan KJ, Cowan CSM, Sandhu KV, Bastiaanssen TFS, Boehme M, Codagnone MG, Cussotto S, Fulling C, Golubeva AV, et al. The microbiota-gut-brain axis. Physiol Rev. 2019;99(4):1877–24. doi:10.1152/physrev.00018.2018. 31460832

[2] Feigin VL, Nichols E, Alam T, Bannick MS, Beghi E, Blake N, Culpepper WJ, Dorsey ER, Elbaz A, Ellenbogen RG, et al. Global, regional, and national burden of neurological disorders, 1990–2016: a systematic analysis for the Global Burden of Disease Study 2016. Lancet Neurol. 2019;18(5):459–480. doi:10.1016/S1474-4422(18)30499-X. 30879893 PMC6459001

[3] Alkasir R, Li J, Li X, Jin M, Zhu B. Human gut microbiota: the links with dementia development. Protein Cell. 2017;8(2):90–102. doi:10.1007/s13238-016-0338-6. 27866330 PMC5291774

[4] Vogt NM, Kerby RL, Dill-McFarland KA, Harding SJ, Merluzzi AP, Johnson SC, Carlsson CM, Asthana S, Zetterberg H, Blennow K, et al. Gut microbiome alterations in Alzheimer’s disease. Sci Rep. 2017;7(1):13537. doi:10.1038/s41598-017-13601-y. 29051531 PMC5648830

[5] Hill‐Burns EM, Debelius JW, Morton JT, Wissemann WT, Lewis MR, Wallen ZD, Peddada SD, Factor SA, Molho E, Zabetian CP, et al. Parkinson’s disease and Parkinson’s disease medications have distinct signatures of the gut microbiome. Mov Disord. 2017;32(5):739–749. doi:10.1002/mds.26942. 28195358 PMC5469442

[6] Jiang H, Ling, Z, Zhang, Y, Mao, H, Ma, Z, Yin, Y, Wang, W, Tang, W, Tan, Z, Shi, J, Li, L, Ruan, B. Altered fecal microbiota composition in patients with major depressive disorder. Brain Behav Immun. 2015;48:186–194. 25882912 10.1016/j.bbi.2015.03.016

[7] Mayneris-Perxachs J, Castells-Nobau, A, Arnoriaga-Rodríguez, M, Martin, M, de la Vega-Correa, L, Zapata, C, Burokas, A, Blasco, G, Coll, C, Escrichs, A, Biarnés, C, Moreno-Navarrete, JM, Puig, J, Garre-Olmo, J, Ramos, R, Pedraza, S, Brugada, R, Vilanova, JC, Serena, J, Gich, J, Ramió-Torrentà, Ll, et al. Microbiota alterations in proline metabolism impact depression. Cell Metab. 2022;34:681–701.e10. 35508109 10.1016/j.cmet.2022.04.001

[8] Gkougka D, Mitropoulos K, Tzanakaki G, Panagouli E, Psaltopoulou T, Thomaidis L, Tsolia M, Sergentanis TN, Tsitsika A. Gut microbiome and attention deficit/hyperactivity disorder: a systematic review. Pediatr Res. 2022;92(6):1507–1519. doi:10.1038/s41390-022-02027-6. 35354932

[9] Strati F, Cavalieri D, Albanese D, De Felice C, Donati C, Hayek J, Jousson O, Leoncini S, Renzi D, Calabrò A, et al. New evidences on the altered gut microbiota in autism spectrum disorders. Microbiome. 2017;5(1):24. doi:10.1186/s40168-017-0242-1. 28222761 PMC5320696

[10] Kang D-W, Ilhan ZE, Isern NG, Hoyt DW, Howsmon DP, Shaffer M, Lozupone CA, Hahn J, Adams JB, Krajmalnik-Brown R. Differences in fecal microbial metabolites and microbiota of children with autism spectrum disorders. Anaerobe. 2018;49:121–131. doi:10.1016/j.anaerobe.2017.12.007. 29274915

[11] Pan Q, Li, YQ, Guo, K, Xue, M, Gan, Y, Wang, K, Xu, DB, Tu, QY. Elderly Patients with Mild Cognitive Impairment Exhibit Altered Gut Microbiota Profiles. J Immunol Res. 2021;2021:1–7. 10.1155/2021/5578958 PMC8635943 34869782

[12] Agustí A, García-Pardo, MP, López-Almela, I, Campillo, I, Maes, M, Romaní-Pérez, M, Sanz, Y. Interplay between the gut-brain axis, obesity and cognitive function. Front Neurosci. 2018;12 155. 29615850 10.3389/fnins.2018.00155 PMC5864897

[13] Arnoriaga-Rodríguez M, Mayneris-Perxachs J, Burokas A, Contreras-Rodríguez O, Blasco G, Coll C, Biarnés C, Miranda-Olivos R, Latorre J, Moreno-Navarrete J-M, et al. Obesity impairs short-term and working memory through gut microbial metabolism of aromatic amino acids. Cell Metab. 2020;32(4):548–560.e7. doi:10.1016/j.cmet.2020.09.002. 33027674

[14] Didari T, Solki S, Mozaffari S, Nikfar S, Abdollahi M. A systematic review of the safety of probiotics. Expert Opin Drug Saf. 2014;13:227–239. 24405164 10.1517/14740338.2014.872627

[15] Mahnert A, Blohs M, Pausan M-R, Moissl-Eichinger C, Robinson NP. The human archaeome: methodological pitfalls and knowledge gaps. Emerg Top Life Sci. 2018;2(4):469–482. doi:10.1042/ETLS20180037. 33525835

[16] Dridi B, Henry M, El Khéchine A, Raoult D, Drancourt M, Dobrindt U. High prevalence of methanobrevibacter smithii and methanosphaera stadtmanae detected in the human gut Using an Improved DNA detection protocol. PLOS ONE. 2009;4(9):e7063. doi:10.1371/journal.pone.0007063. 19759898 PMC2738942

[17] Chibani CM, Mahnert A, Borrel G, Almeida A, Werner A, Brugère J-F, Gribaldo S, Finn RD, Schmitz RA, Moissl-Eichinger C. A catalogue of 1,167 genomes from the human gut archaeome. Nat Microbiol. 2021;7(1):48–61. doi:10.1038/s41564-021-01020-9. 34969981 PMC8727293

[18] Brugère J-F, Borrel G, Gaci N, Tottey W, O’Toole PW, Malpuech-Brugère C. Archaebiotics. Gut Microbes. 2014;5(1):5–10. doi:10.4161/gmic.26749. 24247281 PMC4049937

[19] Chatterjee S, Park S, Low K, Kong Y, Pimentel M. The degree of breath methane production in IBS correlates with the severity of constipation. Am J Gastroenterol. 2007;102(4):837–841. doi:10.1111/j.1572-0241.2007.01072.x. 17397408

[20] Attaluri A, Jackson M, Valestin J, Rao SS. Methanogenic flora is associated with altered colonic transit but not stool characteristics in constipation without IBS. Am J Gastroenterol. 2010;105(6):1407–1411. doi:10.1038/ajg.2009.655. 19953090 PMC3822765

[21] Coker OO, Wu WKK, Wong SH, Sung JJY, Yu J. Altered gut archaea composition and interaction with bacteria are associated with colorectal cancer. gastroenterology. 2020;159(4):1459–1470.e5. doi:10.1053/j.gastro.2020.06.042. 32569776

[22] Camara A, Konate S, Tidjani Alou M, Kodio A, Togo AH, Cortaredona S, Henrissat B, Thera MA, Doumbo OK, Raoult D, et al. Clinical evidence of the role of Methanobrevibacter smithii in severe acute malnutrition. Sci Rep. 2021;11(1):5426. doi:10.1038/s41598-021-84641-8. 33686095 PMC7940396

[23] Basseri RJ, Basseri, B, Pimentel, M, Chong, K, Youdim, A, Low, K, Hwang, L, Soffer, E, Chang, C, Mathur, R. Intestinal methane production in obese individuals is associated with a higher body mass index. Gastroenterol Hepatol. 2012;8:22–28. PMC3277195 22347829

[24] Wilder-Smith CH, Olesen SS, Materna A, Drewes AM. Breath methane concentrations and markers of obesity in patients with functional gastrointestinal disorders. U Eur Gastroenterol J. 2018;6(4):595–603. doi:10.1177/2050640617744457. PMC5987281 29881615

[25] Mbakwa CA, Penders, J, Savelkoul, PH, Thijs, C, Dagnelie, PC, Mommers, M, Arts, ICW. Gut colonization with methanobrevibacter smithii is associated with childhood weight development. Obesity. 2015;23:2508–2516. 26524691 10.1002/oby.21266

[26] Zhang H, DiBaise, JK, Zuccolo, A, Kudrna, D, Braidotti, M, Yu, Y, Parameswaran, P, Crowell, MD, Wing, R, Rittmann, BE, Krajmalnik-Brown, R. Human gut microbiota in obesity and after gastric bypass. Proc Natl Acad Sci. 2009;106:2365–2370. 19164560 10.1073/pnas.0812600106 PMC2629490

[27] Armougom F, Henry M, Vialettes B, Raccah D, Raoult D, Ratner AJ. Monitoring bacterial community of human gut microbiota reveals an increase in lactobacillus in obese patients and methanogens in anorexic patients. PLOS ONE. 2009;4(9):e7125. doi:10.1371/journal.pone.0007125. 19774074 PMC2742902

[28] Hua S, Peters BA, Lee S, Fitzgerald K, Wang Z, Sollecito CC, Grassi E, Wiek F, St Peter L, D’Souza G, et al. Gut microbiota and cognitive function among women living with HIV. J Alzheimers Dis. 2023;95(3):1147–1161. doi:10.3233/JAD-230117. 37661881 PMC10771810

[29] Misiak B, Pawlak E, Rembacz K, Kotas M, Żebrowska-Różańska P, Kujawa D, Łaczmański Ł, Piotrowski P, Bielawski T, Samochowiec J, et al. Associations of gut microbiota alterations with clinical, metabolic, and immune-inflammatory characteristics of chronic schizophrenia. J Psychiatr Res. 2024;171:152–160. doi:10.1016/j.jpsychires.2024.01.036. 38281465

[30] Jangi S, Gandhi R, Cox LM, Li N, von Glehn F, Yan R, Patel B, Mazzola MA, Liu S, Glanz BL, et al. Alterations of the human gut microbiome in multiple sclerosis. Nat Commun. 2016;7(1):12015. doi:10.1038/ncomms12015. 27352007 PMC4931233

[31] Djemai K, Drancourt M, Tidjani Alou M. Bacteria and Methanogens in the Human Microbiome: a Review of Syntrophic Interactions. Microb Ecol. 2022;83(3):536–554. doi:10.1007/s00248-021-01796-7. 34169332

[32] STROOP GCJ. Test de Colores y Palabras—Edición Revisada. 2020.

[33] Wechsler D, de la Guía E, Vallar F. WAIS-IV: Escala de Inteligencia de Wechsler Para Adultos-IV. Madrid: Pearson. 2012.

[34] Duller S, Vrbancic, S, Szydłowski, Ł, Mahnert, A, Blohs, M, Predl, M, Kumpitsch, C, Zrim, V, Högenauer, C, Kosciolek, T, Schmitz, RA, Eberhard, A, Dragovan, M, Schmidberger, L, Zurabischvili, T, Weinberger, V, Moser, AM, Kolb, D, Pernitsch, D, Mohammadzadeh, R, Kühnast, T, et al. Targeted isolation of Methanobrevibacter strains from fecal samples expands the cultivated human archaeome. Nat Commun. 2024;15:7593. 39217206 10.1038/s41467-024-52037-7 PMC11366006

[35] Mafra D, Ribeiro M, Fonseca L, Regis B, Cardozo LFMF, Fragoso dos Santos H, Emiliano de Jesus H, Schultz J, Shiels PG, Stenvinkel P, et al. Archaea from the gut microbiota of humans: Could be linked to chronic diseases?. Anaerobe. 2022;77:102629. doi:10.1016/j.anaerobe.2022.102629. 35985606

[36] Bui TPN, Schols HA, Jonathan M, Stams AJM, de Vos WM, Plugge CM. Mutual Metabolic Interactions in Co-cultures of the Intestinal Anaerostipes rhamnosivorans With an Acetogen, Methanogen, or Pectin-Degrader Affecting Butyrate Production. Front Microbiol. 2019;10. doi:10.3389/fmicb.2019.02449. PMC6839446 31736896

[37] Li Y, Liu A, Chen K, Li L, Zhang X, Zou F, Zhang X, Meng X. Sodium butyrate alleviates lead-induced neuroinflammation and improves cognitive and memory impairment through the ACSS2/H3K9ac/BDNF pathway. Environ Int. 2024;184:108479. doi:10.1016/j.envint.2024.108479. 38340407

[38] Ge X, Zheng M, Hu M, Fang X, Geng D, Liu S, Wang L, Zhang J, Guan L, Zheng P, et al. Butyrate ameliorates quinolinic acid–induced cognitive decline in obesity models. J Clin Invest. 2023;133(4). doi:10.1172/JCI154612. PMC9927952 36787221

[39] Liu H, Wang J, He T, Becker S, Zhang G, Li D, Ma X. Butyrate: A Double-Edged Sword for Health?. Adv Nutr. 2018;9(1):21–29. doi:10.1093/advances/nmx009. 29438462 PMC6333934

[40] Zhang L, Liu C, Jiang Q, Yin Y. Butyrate in energy metabolism: There is still more to learn. Trends Endocrinol Metab. 2021;32(3):159–169. doi:10.1016/j.tem.2020.12.003. 33461886

[41] van Deuren T, Blaak EE, Canfora EE. Butyrate to combat obesity and obesity-associated metabolic disorders: Current status and future implications for therapeutic use. Obes Rev Off J Int Assoc Study Obes. 2022;23(10):e13498. doi:10.1111/obr.13498. PMC9541926 35856338

[42] Hong J, Jia Y, Pan S, Jia L, Li H, Han Z, Cai D, Zhao R. Butyrate alleviates high fat diet-induced obesity through activation of adiponectin-mediated pathway and stimulation of mitochondrial function in the skeletal muscle of mice. Oncotarget. 2016;7(35):56071–56082. doi:10.18632/oncotarget.11267. 27528227 PMC5302897

[43] Lv W, Lin, X, Shen, H, Liu, HM, Qiu, X, Shen, WD, Ge, CL, Lv, FY, Shen, J, Xiao, HM, Deng, HW. Human gut microbiome impacts skeletal muscle mass via gut microbial synthesis of the short‐chain fatty acid butyrate among healthy menopausal women. J Cachexia Sarcopenia Muscle. 2021;12:1860–1870. 34472211 10.1002/jcsm.12788 PMC8718076

[44] Long CL, Haverberg LN, Young VR, Kinney JM, Munro HN, Geiger JW. Metabolism of 3-methylhistidine in man. Metabolism. 1975;24(8):929–935. doi:10.1016/0026-0495(75)90084-0. 1143090

[45] Long CL, Dillard DR, Bodzin JH, Geiger JW, Blakemore WS. Validity of 3-methylhistidine excretion as an indicator of skeletal muscle protein breakdown in humans. Metabolism. 1988;37:844–849. 3138511 10.1016/0026-0495(88)90118-7

[46] Dora K, Tsukamoto H, Suga T, Tomoo K, Suzuki A, Adachi Y, Takeshita M, Kato Y, Kawasaki M, Sato W, et al. Essential amino acid supplements ingestion has a positive effect on executive function after moderate-intensity aerobic exercise. Sci Rep. 2023;13(1):22644. doi:10.1038/s41598-023-49781-z. 38114553 PMC10730626

[47] Yamada T, Kurasawa S, Matsuzaki M, Tanaka A. Body weight reduction by exercise increases the urinary 3-methylhistidine excretion level with relatively positive nitrogen, sodium, and potassium balances when compared to dietary restriction. Heliyon. 2023;9:e19632. 37809975 10.1016/j.heliyon.2023.e19632 PMC10558883

[48] Petersen LM, Bautista EJ, Nguyen H, Hanson BM, Chen L, Lek SH, Sodergren E, Weinstock GM. Community characteristics of the gut microbiomes of competitive cyclists. Microbiome. 2017;5(1). doi:10.1186/s40168-017-0320-4. PMC5553673 28797298

[49] Sui SX, Williams LJ, Holloway-Kew KL, Hyde NK, Pasco JA. Skeletal Muscle Health and Cognitive Function: A Narrative Review. Int J Mol Sci. 2020;22(1):255. doi:10.3390/ijms22010255. 33383820 PMC7795998

[50] Zhao J, Huang Y, Yu X. A Narrative Review of Gut-Muscle Axis and Sarcopenia: The Potential Role of Gut Microbiota. Int J Gen Med. 2021;14:1263–1273. doi:10.2147/IJGM.S301141. 33880058 PMC8053521

[51] Ostfeld I, Hoffman JR. The Effect of β-Alanine Supplementation on Performance, Cognitive Function and Resiliency in Soldiers. Nutrients. 2023;15(4):1039. doi:10.3390/nu15041039. 36839397 PMC9961614

[52] Song J, Yang L, Nan D, He Q, Wan Y, Guo H. Histidine Alleviates Impairments Induced by Chronic Cerebral Hypoperfusion in Mice. Front Physiol. 2018;9. doi:10.3389/fphys.2018.00662. PMC5999792 29930513

[53] Nakamura T, Naganuma, F, Kudomi, U, Roh, S, Yanai, K, Yoshikawa, T. Oral histidine intake improves working memory through the activation of histaminergic nervous system in mice. Biochem Biophys Res Commun. 2022;609:141–148. 35429681 10.1016/j.bbrc.2022.04.016

[54] Cerdó T, Ruiz-Rodríguez A, Acuña I, Torres-Espínola FJ, Menchén-Márquez S, Gámiz F, Gallo M, Jehmlich N, Haange S-B, von Bergen M, et al. Infant gut microbiota contributes to cognitive performance in mice. Cell Host & Microbe. 2023;31(12):1974–1988.e4. doi:10.1016/j.chom.2023.11.004. 38052208

[55] Balleine BW, Delgado MR, Hikosaka O. The role of the dorsal striatum in reward and decision-making: figure 1. J Neurosci Off J Soc Neurosci. 2007;27(31):8161–8165. doi:10.1523/JNEUROSCI.1554-07.2007. PMC6673072 17670959

[56] Ravizza SM, Goudreau J, Delgado MR, Ruiz S. Executive function in Parkinson’s disease: contributions of the dorsal frontostriatal pathways to action and motivation. Cogn Affect Behav Neurosci. 2012;12:193–206. 22006555 10.3758/s13415-011-0066-6

[57] Zhu H, Wang N, Yao L, Chen Q, Zhang R, Qian J, Hou Y, Guo W, Fan S, Liu S, et al. Moderate uv exposure enhances learning and memory by promoting a novel glutamate biosynthetic pathway in the brain. Cell. 2018;173(7):1716–1727.e17. doi:10.1016/j.cell.2018.04.014. 29779945

[58] MahmoudianDehkordi S, Arnold M, Nho K, Ahmad S, Jia W, Xie G, Louie G, Kueider‐Paisley A, Moseley MA, Thompson JW, et al. Altered bile acid profile associates with cognitive impairment in Alzheimer’s disease—an emerging role for gut microbiome. Alzheimers & Dementia. 2019;15(1):76–92. doi:10.1016/j.jalz.2018.07.217. PMC6487485 30337151

[59] Baloni P, Funk CC, Yan J, Yurkovich JT, Kueider-Paisley A, Nho K, Heinken A, Jia W, Mahmoudiandehkordi S, Louie G, et al. Metabolic Network Analysis Reveals Altered Bile Acid Synthesis and Metabolism in Alzheimer’s Disease. Cell Rep Med. 2020;1(8):100138. doi:10.1016/j.xcrm.2020.100138. 33294859 PMC7691449

[60] Jiang Z, Zhuo L-B, He Y, Fu Y, Shen L, Xu F, Gou W, Miao Z, Shuai M, Liang Y, et al. The gut microbiota-bile acid axis links the positive association between chronic insomnia and cardiometabolic diseases. Nat Commun. 2022;13(1):3002. doi:10.1038/s41467-022-30712-x. 35637254 PMC9151781

[61] Lee AM, Xu Y, Hooper SR, Abraham AG, Hu J, Xiao R, Matheson MB, Brunson C, Rhee EP, Coresh J, et al. Circulating metabolomic associations with neurocognitive outcomes in pediatric ckd. Clin J Am Soc Nephrol CJASN. 2024;19(1):13–25. doi:10.2215/CJN.0000000000000318. 37871960 PMC10843217

[62] Kurella Tamura M, Chertow, GM, Depner, TA, Nissenson, AR, Schiller, B, Mehta, RL, Liu, S, Sirich, TL, FNH, Study. Metabolic Profiling of Impaired Cognitive Function in Patients Receiving Dialysis. J Am Soc Nephrol JASN. 2016;27:3780–3787. 27444566 10.1681/ASN.2016010039 PMC5118491

[63] Yang J, Zhou Y, Wang T, Li N, Chao Y, Gao S, Zhang Q, Wu S, Zhao L, Dong X. A multi-omics study to monitor senescence-associated secretory phenotypes of Alzheimer’s disease. Ann Clin Transl Neurol. 2024;11(5):1310–1324. doi:10.1002/acn3.52047. 38605603 PMC11093245

[64] Grande de França NA, Díaz G, Lengelé L, Soriano G, Caspar-Bauguil S, Saint-Aubert L, Payoux P, Rouch L, Vellas B, de Souto Barreto P, et al. Associations between blood nutritional biomarkers and cerebral amyloid-β: insights from the cogfrail cohort study. J Gerontol A Biol Sci Med Sci. 2024;79(1):glad248. doi:10.1093/gerona/glad248. 37879623

[65] Corrigan FM, Horrobin DF, Skinner ER, Besson JA, Cooper MB. Abnormal content of n−6 and n−3 long-chain unsaturated fatty acids in the phosphoglycerides and cholesterol esters of parahippocampal cortex from Alzheimer’s disease patients and its relationship to acetyl CoA content. Int J Biochem Cell Biol. 1998;30(2):197–207. doi:10.1016/S1357-2725(97)00125-8. 9608673

[66] Guan Z, Wang, Y, Cairns, NJ, Lantos, PL, Dallner, G, Sindelar, PJ. Decrease and structural modifications of phosphatidylethanolamine plasmalogen in the brain with Alzheimer disease. J Neuropathol Exp Neurol. 1999;58:740–747. 10411344 10.1097/00005072-199907000-00008

[67] Dalile B, Van Oudenhove L, Vervliet B, Verbeke K. The role of short-chain fatty acids in microbiota–gut–brain communication. Nat Rev Gastroenterol Hepatol. 2019;16(8):461–478. doi:10.1038/s41575-019-0157-3. 31123355

[68] Puig J, Biarnes C, Pedraza S, Vilanova JC, Pamplona R, Fernández-Real JM, Brugada R, Ramos R, Coll-de-Tuero G, Calvo-Perxas L, et al. The aging imageomics study: rationale, design and baseline characteristics of the study population. Mech Ageing Dev. 2020;189:111257. doi:10.1016/j.mad.2020.111257. 32437737

[69] Chen S, Zhou Y, Chen Y, Gu J. fastp: an ultra-fast all-in-one FASTQ preprocessor. Bioinformatics. 2018;34(17):i884–i890. doi:10.1093/bioinformatics/bty560. 30423086 PMC6129281

[70] Langmead B, Salzberg SL. Fast gapped-read alignment with Bowtie 2. Nat Methods. 2012;9(4):357–359. doi:10.1038/nmeth.1923. 22388286 PMC3322381

[71] Tamames J, Puente-Sánchez FS. A highly portable, fully automatic metagenomic analysis pipeline. Front Microbiol. 2019;9:3349. 30733714 10.3389/fmicb.2018.03349 PMC6353838

[72] Li D, Liu C-M, Luo R, Sadakane K, Lam T-W. MEGAHIT: an ultra-fast single-node solution for large and complex metagenomics assembly via succinct de Bruijn graph. Bioinformatics. 2015;31(10):1674–1676. doi:10.1093/bioinformatics/btv033. 25609793

[73] Hyatt D, Chen, GL, LoCascio, PF, Land, ML, Larimer, FW, Hauser, LJ. Prodigal: prokaryotic gene recognition and translation initiation site identification. BMC Bioinf. 2010;11:119. 10.1186/1471-2105-11-119 PMC2848648 20211023

[74] Buchfink B, Reuter K, Drost H-G. Sensitive protein alignments at tree-of-life scale using DIAMOND. Nat Methods. 2021;18:366–368. 33828273 10.1038/s41592-021-01101-x PMC8026399

[75] Wikoff WR, Pendyala G, Siuzdak G, Fox HS. Metabolomic analysis of the cerebrospinal fluid reveals changes in phospholipase expression in the CNS of SIV-infected macaques. J Clin Invest. 2008;118, JCI34138 (7). doi:10.1172/JCI34138. PMC2398736 18521184

[76] Sarafian MH, Lewis MR, Pechlivanis A, Ralphs S, McPhail MJW, Patel VC, Dumas M-E, Holmes E, Nicholson JK. Bile Acid Profiling and Quantification in Biofluids Using Ultra-Performance Liquid Chromatography Tandem Mass Spectrometry. Anal Chem. 2015;87(19):9662–9670. doi:10.1021/acs.analchem.5b01556. 26327313

[77] Lin H, Peddada SD. Analysis of compositions of microbiomes with bias correction. Nat Commun. 2020;11(1). doi:10.1038/s41467-020-17041-7. PMC7360769 32665548

[78] Carvajal-Rodríguez A, de Uña-Alvarez J, Rolán-Alvarez E. A new multitest correction (SGoF) that increases its statistical power when increasing the number of tests. BMC Bioinf. 2009;10:209. 10.1186/1471-2105-10-209 PMC2719628 19586526

[79] Wu T, Hu E, Xu S, Chen M, Guo P, Dai Z, Feng T, Zhou L, Tang W, Zhan L, et al. clusterProfiler 4.0: A universal enrichment tool for interpreting omics data. The Innov. 2021;2(3):100141. doi:10.1016/j.xinn.2021.100141. PMC8454663 34557778

[80] Storey JD. A Direct Approach to False Discovery Rates. J R Statist Soc B. 2002;64(3):479–498. doi:10.1111/1467-9868.00346.

[81] Peschel S, Müller CL, Von Mutius E, Boulesteix AL, Depner M. NetCoMi: network construction and comparison for microbiome data in R. Brief Bioinformat. 2021;22(4):bbaa290. doi:10.1093/bib/bbaa290. PMC8293835 33264391

[82] Kursa MB, Rudnicki WR. Feature Selection with the Boruta Package. JSS J Stat Softw. 2010;36(11). http://www.jstatsoft.org/.

[83] Lundberg SM, Lee S-I. A Unified Approach to Interpreting Model Predictions (eds. Advances in Neural Information Processing Systems. Vol. 30 Guyon, I, Bengio, S, Wallach, H, Fergus, R, Vishwanathan, S, Garnett, R. Red Hook, New York, USA.: Curran Associates, Inc.; 2017 4765–4774.

[84] Ritchie ME, Phipson B, Wu D, Hu Y, Law CW, Shi W, Smyth GK. limma powers differential expression analyses for RNA-sequencing and microarray studies. Nucleic Acids Res. 2015;43(7):e47–e47. doi:10.1093/nar/gkv007. 25605792 PMC4402510

