# Supplementary material for: Archaea methanogens are associated with cognitive performance through the shaping of gut microbiota, butyrate and histidine metabolism
Source: Gut Microbes. 2025 Feb 5;17(1):2455506. doi: 10.1080/19490976.2025.2455506 (PMC11810085; doi:10.1080/19490976.2025.2455506)
Supplement: Supplemental Material [file KGMI_A_2455506_SM1706.docx]

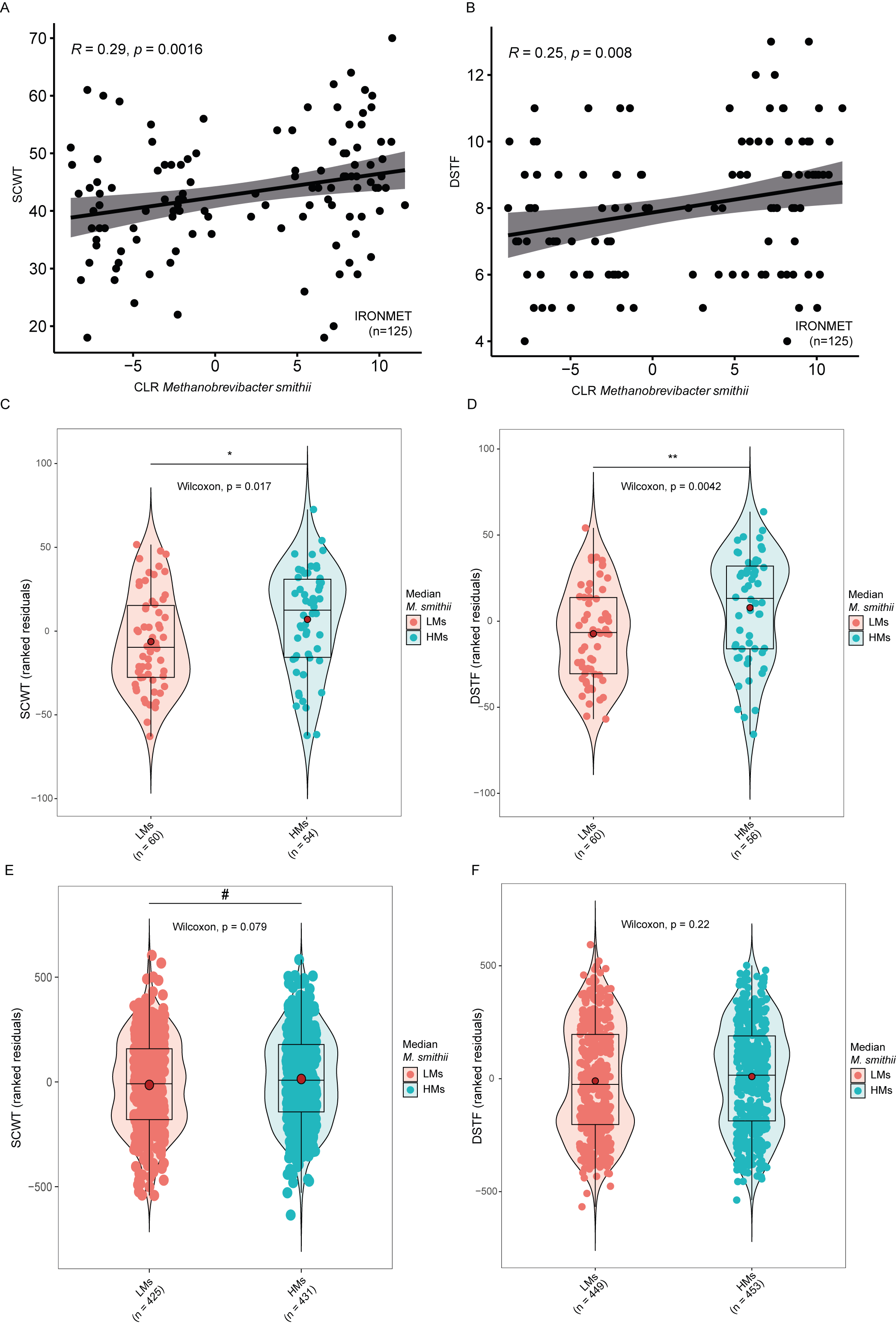
**Figure S1. Adjusted cognitive scores associated to *M. smithii* groups.** Scatter plot of the Spearman’s correlation between SCWT-CW (**A**) and DSTF (**B**) tests scores and CLR-transformed *M.smithii* in the IRONMET cohort Violin plots of the SCWT-CW (**C**) and DSTF (**D**) tests scores in the IRONMET cohort grouped according to LMs-HMs group after controlling for age, sex, BMI, and education years. The ranked residuals are plotted. Violin plots of the SCWT-CW (**E**) DSTF (**F**) tests scores in the Aging Imageomics cohort grouped according to LMs-HMs group after controlling for age, sex, BMI, and education years. The ranked residuals are plotted. Significance was assessed using a Wilcoxon test. Red dots represent the mean. #p<0.1 *p<0.05, **p<0.01; ***p<0.001.


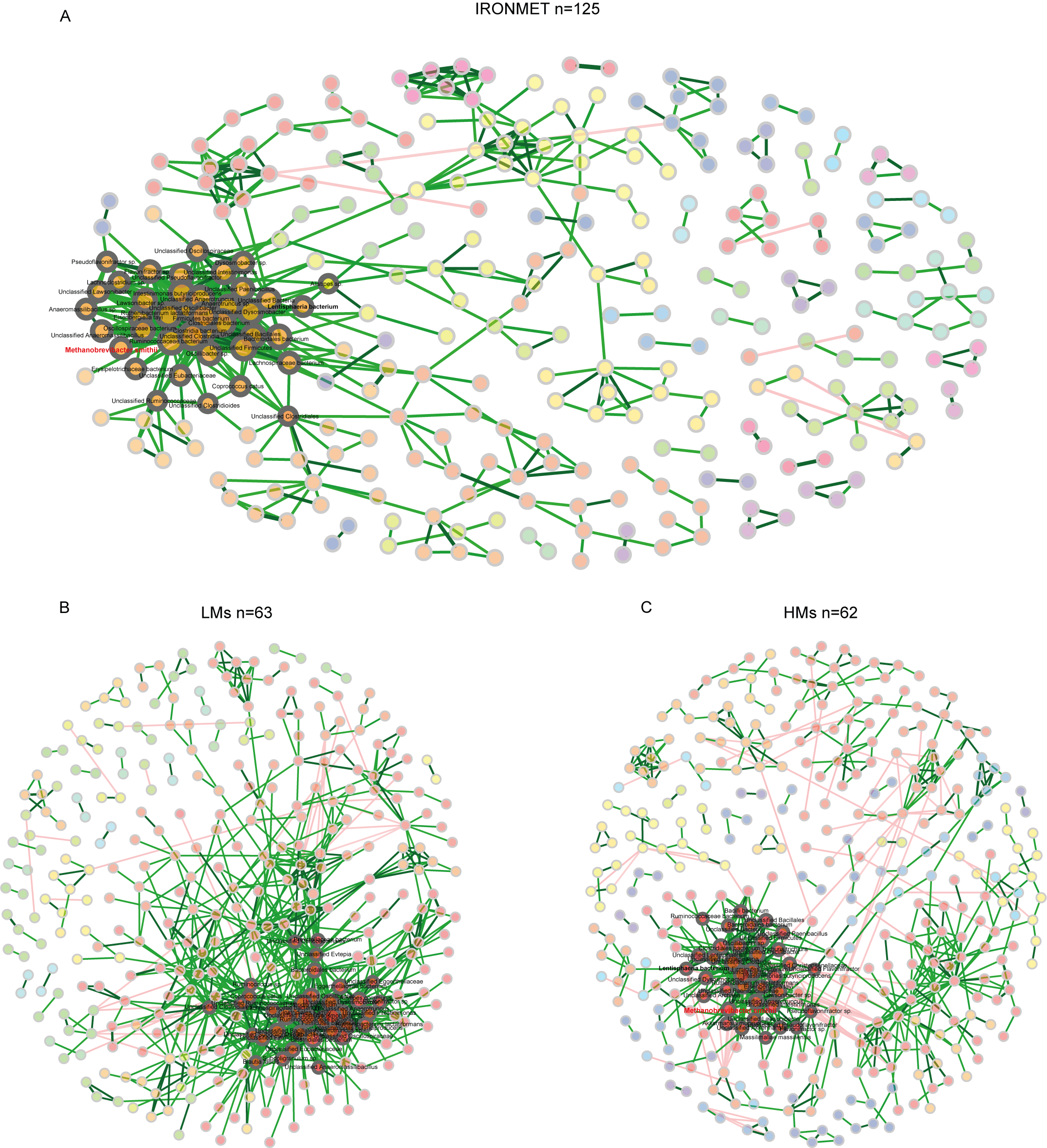
**Figure S2. Network of interspecies associations.** Spearman’s correlation was employed as the measure of association. Partial correlations were transformed into dissimilarities using the “signed” distance metric, with the resulting non-negative similarities serving as edge weights. Positive associations are represented by green edges, while negative associations are depicted in red. Hubs—nodes with centrality values above the empirical 95th percentile—are identified by bold text and borders, with eigenvector centrality used to define their significance and scale their sizes. Node colors indicate clusters, determined through greedy modularity optimization. (**A**) complete network, (**B**) LMs group and (**C**) HMs group for the IRONMET cohort. Unconnected nodes are removed. Hub-nodes species names are reported, *M. smithii* is highlighted in red.


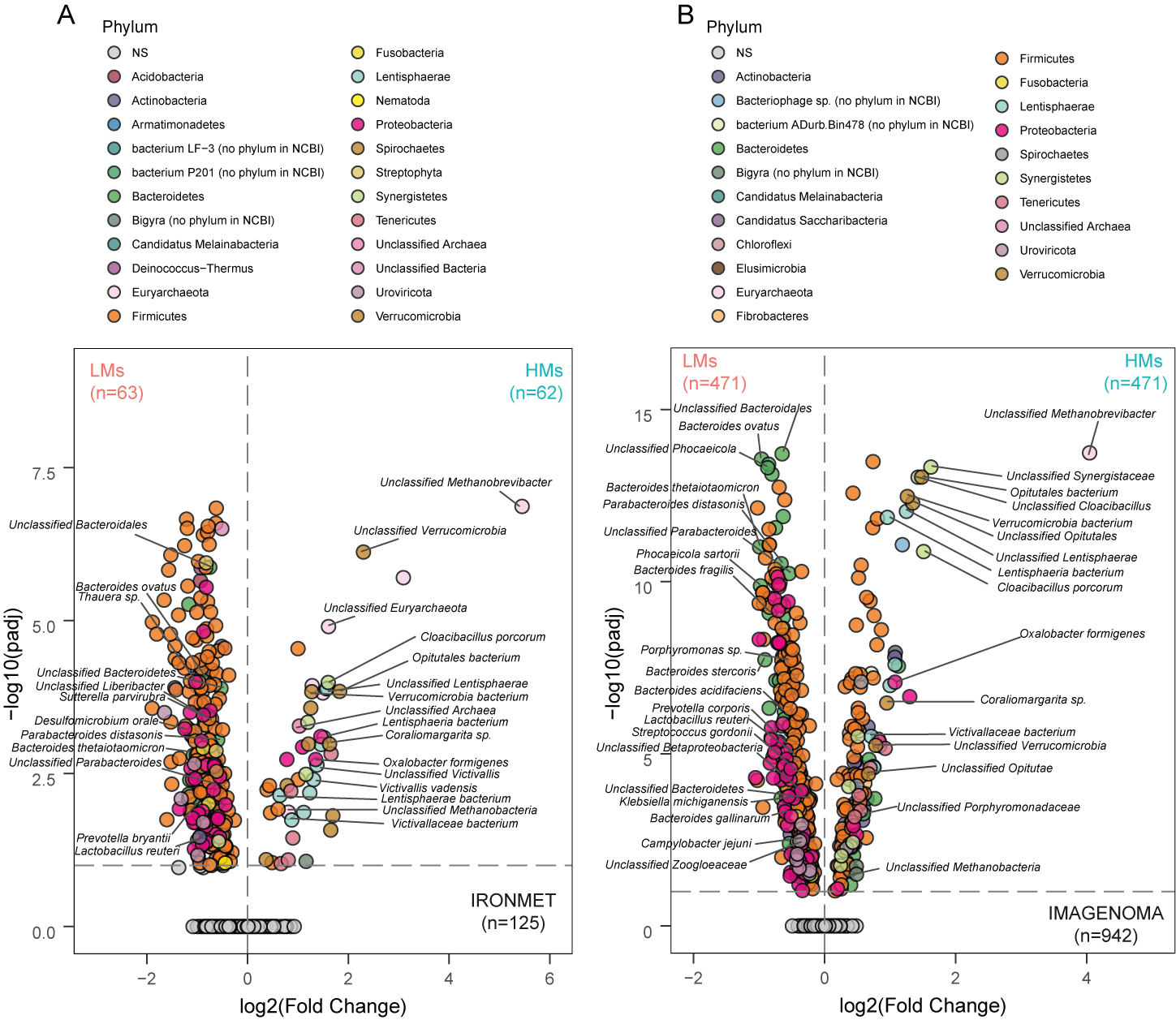
**Figure S3. Gut microbial profile associated with *M. smithii* groups.**

Volcano plot of differential microbial abundance associated with LMs-HMs groups in (**A**) IRONMET and (**B**) Aging Imageomics cohort. Significant species were identified using the ANCOM-BC from shotgun metagenomics data adjusted for age, sex, BMI, years of education and insulin levels. The log2 fold change of the association with a unit change in the ANCOM-BC-transformed variable values and the log10 p values adjusted for multiple comparisons using a sequential goodness of fit were plotted for each taxon. Significantly different taxa are coloured according to phylum. Significance was set at padj<0.1.(**E**)


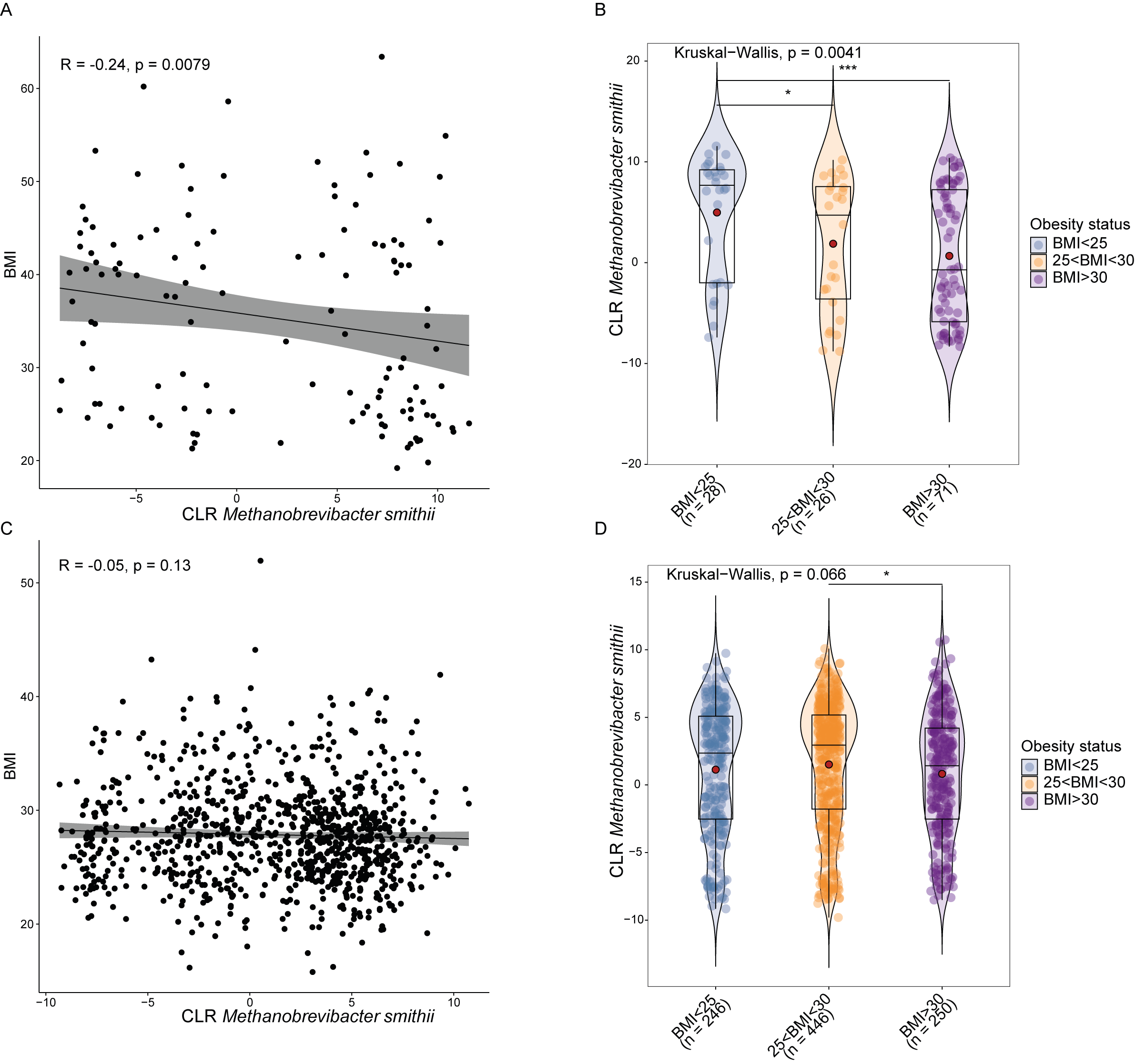


**Figure S4. CLR-transformed *M. smithii* abundance correlation with obesity status.** Spearman correlation between centred log ratio (CLR) of the abundances of *Methanobrevibacter smithii* and the BMI in IRONMET cohort (**A**) and in Aging Imageomics cohort (**C**). Violin plots of the CLR of *M. smithii* grouped according to the obesity status in IRONMET cohort (**B**) and in Aging Imageomics cohort (**D**). Overall significance was assessed using a Kruskal-Wallis test while significance between groups was assessed using a Wilcoxon test. Red dots represent the mean. #p<0.1 *p<0.05, **p<0.01; ***p<0.001.


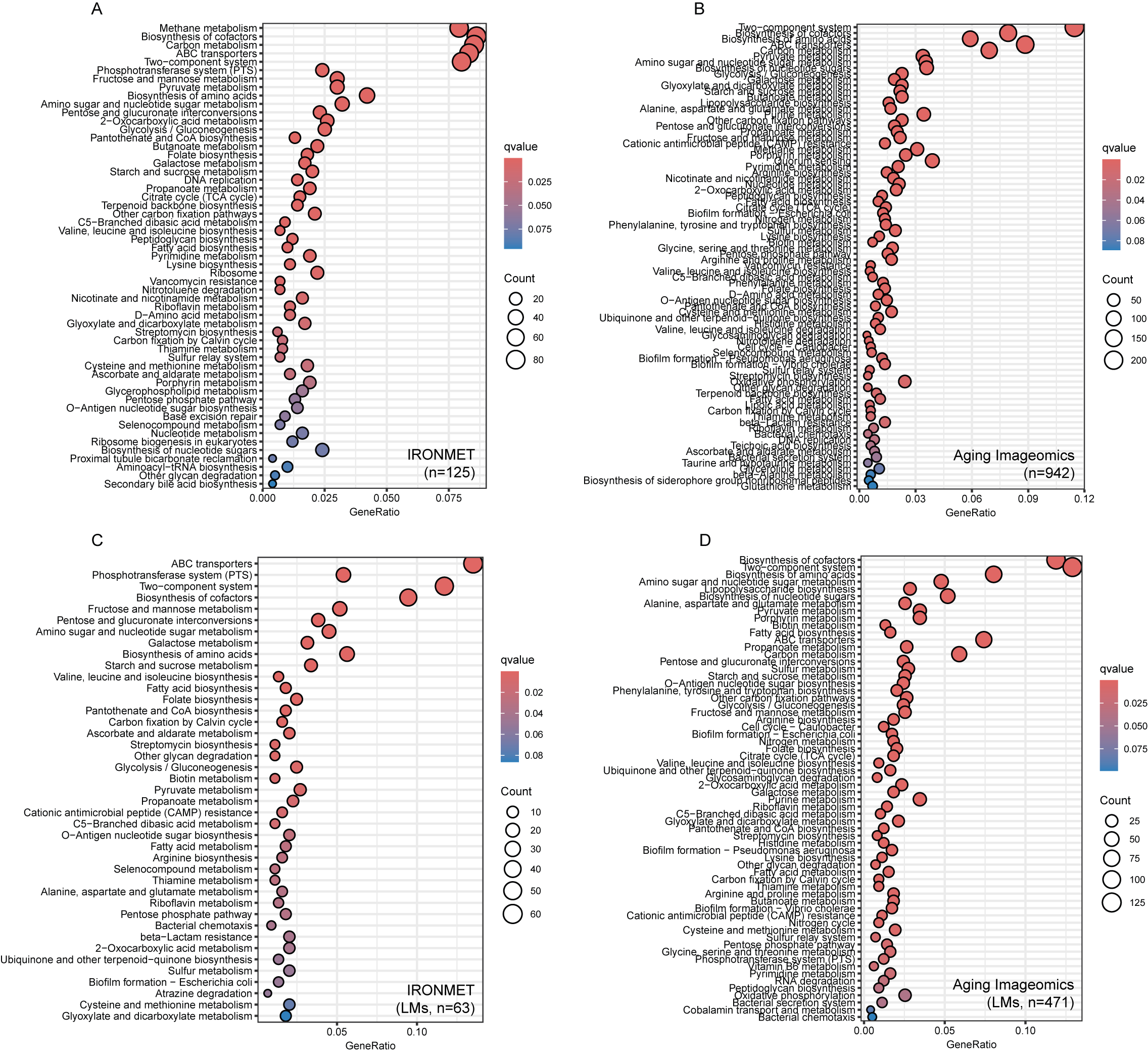


**Figure S5. Functional profile associated with *M. smithii* groups.** Dotplot of KEGG enriched pathways (*q-*value <0.1) from significantly differentially expressed microbial microbial molecular functions associated with the the LMs-HMs group in IRONMET cohort (**A**) and in Aging Imageomics cohort (**B**), and LMs group in IRONMET cohort (**C**) and in Aging Imageomics cohort (**D**). Significant KEGG orthologues were identified by ANCOM-BC after adjusting for age, sex, BMI and years of education. Dots are coloured according to the *q*-value. Common pathways are highlighted.


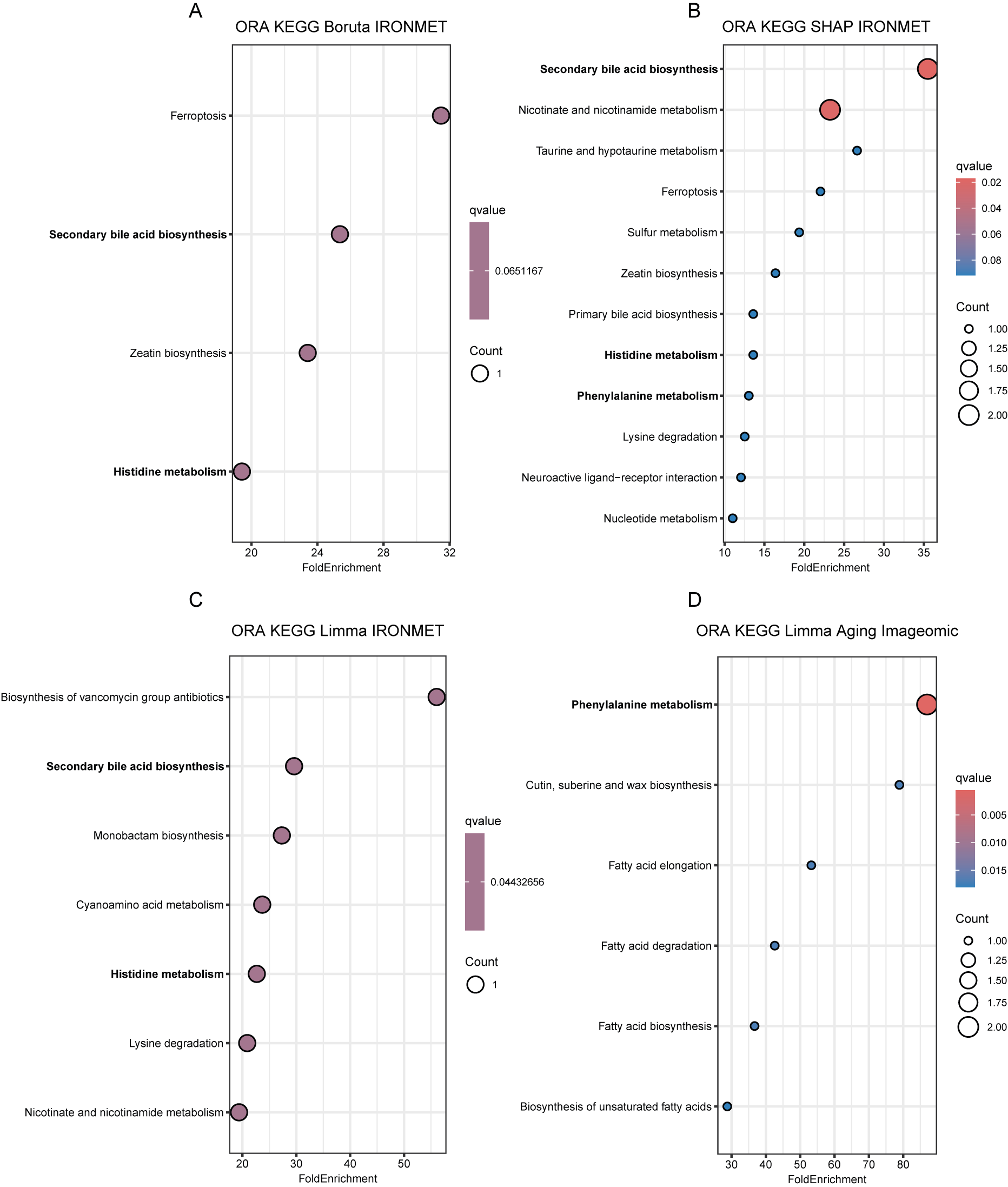


**Figure S6. KEGG-based Over-representation analysis in human cohorts.** Dotplot of KEGG-based Over-representation analysis using the metabolites associated with LMs-HMs groups identified with Boruta (A), SHAP (B), and Limma (C) in IRONMET cohort, and in Aging Imageomics cohort, identified with Limma (D).


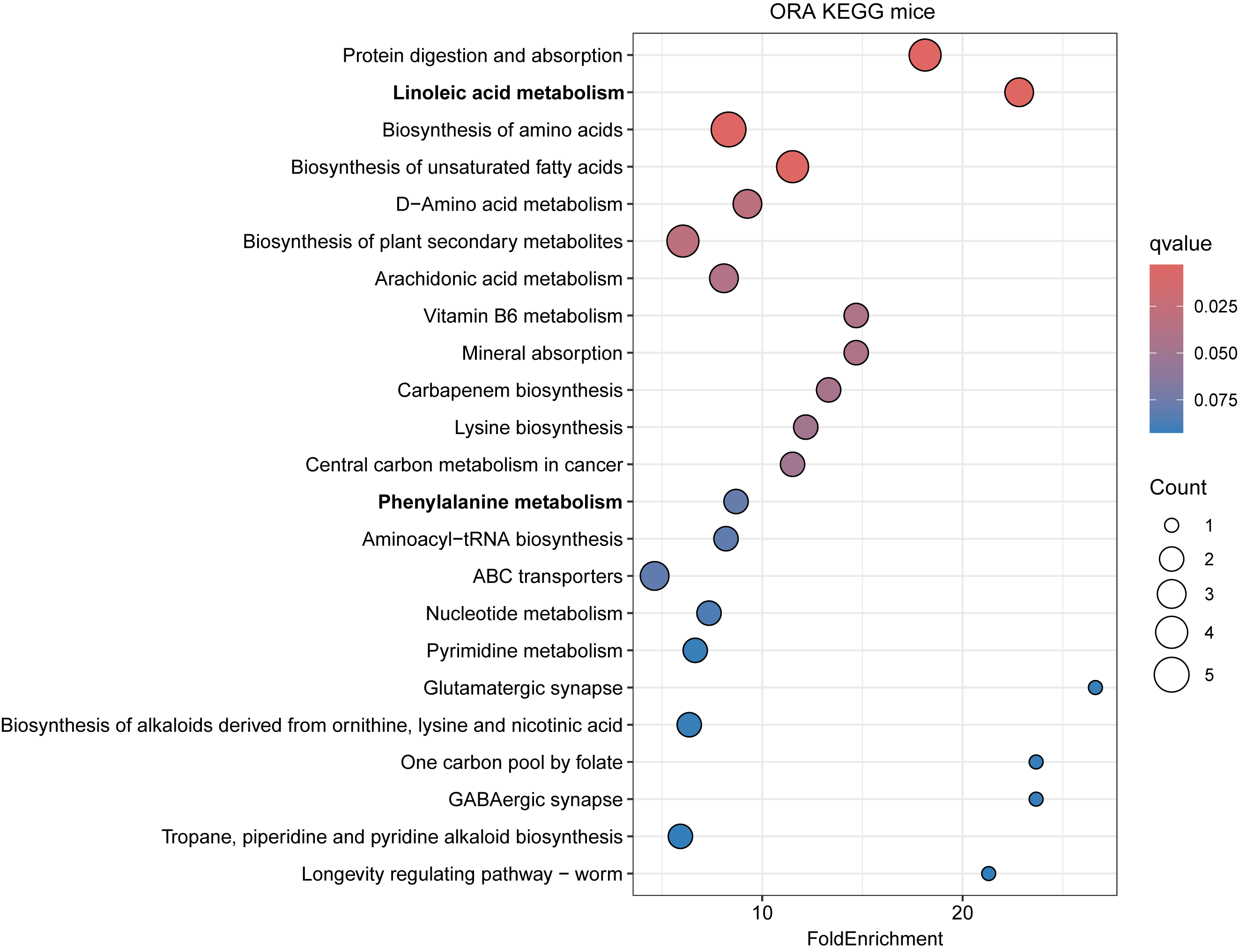


**Figure S7. KEGG-based Over-representation analysis in mice.** Dotplot of KEGG-based Over-representation analysis using the metabolites associated with LMs-HMs groups identified with Limma.
